# Clustered ChIP-Seq-defined transcription factor binding sites and histone modifications map distinct classes of regulatory elements

**DOI:** 10.1186/1741-7007-9-80

**Published:** 2011-11-24

**Authors:** Morten Rye, Pål Sætrom, Tony Håndstad, Finn Drabløs

**Affiliations:** 1 Department of Cancer Research and Molecular Medicine Laboratory Center, Faculty of Medicine, Norwegian University of Science and Technology, Erling Skjalgssons Gate 1, NO-7491 Trondheim, Norway; 2 Department of Computer and Information Science, Sem Sælands Vei 7-9, Norwegian University of Science and Technology, NO-7491 Trondheim, Norway

**Keywords:** transcription factor, ChIP-Seq, histone modification, chromatin

## Abstract

**Background:**

Transcription factor binding to DNA requires both an appropriate binding element and suitably open chromatin, which together help to define regulatory elements within the genome. Current methods of identifying regulatory elements, such as promoters or enhancers, typically rely on sequence conservation, existing gene annotations or specific marks, such as histone modifications and p300 binding methods, each of which has its own biases.

**Results:**

Herein we show that an approach based on clustering of transcription factor peaks from high-throughput sequencing coupled with chromatin immunoprecipitation (Chip-Seq) can be used to evaluate markers for regulatory elements. We used 67 data sets for 54 unique transcription factors distributed over two cell lines to create regulatory element clusters. By integrating the clusters from our approach with histone modifications and data for open chromatin, we identified general methylation of lysine 4 on histone H3 (H3K4me) as the most specific marker for transcription factor clusters. Clusters mapping to annotated genes showed distinct patterns in cluster composition related to gene expression and histone modifications. Clusters mapping to intergenic regions fall into two groups either directly involved in transcription, including miRNAs and long noncoding RNAs, or facilitating transcription by long-range interactions. The latter clusters were specifically enriched with H3K4me1, but less with acetylation of lysine 27 on histone 3 or p300 binding.

**Conclusion:**

By integrating genomewide data of transcription factor binding and chromatin structure and using our data-driven approach, we pinpointed the chromatin marks that best explain transcription factor association with different regulatory elements. Our results also indicate that a modest selection of transcription factors may be sufficient to map most regulatory elements in the human genome.

## Background

Transcription factors are DNA-binding proteins that regulate gene expression by binding to promoter regions proximal to gene transcription start sites (TSSs) or to more distal enhancer regions that regulate expression through long-range interactions [1-3]. Transcription factor binding varies between cell types, and one major factor contributing to this cell type-specific binding is chromatin structure. Chromatin consists of DNA wrapped around nucleosomes, and chains of nucleosomes linked by DNA are organised structurally into different domains of accessible (open) and inaccessible (closed) chromatin [4-7]. Chromatin accessibility is regulated by DNA methylation and posttranslational modifications in the N-terminal tails of the nucleosomal histone proteins. Although there are no known combinations of modifications that delineate accessible and closed chromatins, histone acetylation and mono-, di- and trimethylation of lysine 4 on histone H3 (H3K4me1, H3K4me2 and H3K4me3, respectively) are generally associated with accessible chromatin, whereas H3K9me3 and H3K27me3 are associated with closed chromatin. Several other modifications coexist with these marks over different domains [4], but these modifications are generally less characterised.

Although the interplay between chromatin environments and transcription factor binding is not straightforward, accessible chromatin generally facilitates association of transcription factors to DNA. Some transcription factors, however, can modify the chromatin landscape around their binding site, which may recruit new transcription factors and chromatin-modifying factors to the region [1,4]. Changes brought on by such events are the foundation for cell differentiation, whereby chromatin domains and transcription factor binding can be used as markers for cell type-specific regulation. Recent advances in high-throughput sequencing coupled with chromatin immunoprecipitation (ChIP-Seq) [8,9] have enabled genomewide mapping of such domains. Though several studies have used ChIP-Seq to analyse large sets of transcription factors in different organisms [10,11] or the interplay between sets of histone modifications [7,12-20], few studies have investigated the relationship between large sets of transcription factors and histone modifications [18,21,22]. One reason for this is that such a data set would require considerable resources to produce. However, the joint efforts of researchers in the laboratories participating in the ENCODE project [23] are now making such studies possible in a few selected cell lines.

The goal of this study was twofold. First, we wanted to investigate whether genomic regions enriched with bound transcription factors can be used to improve the identification of regulatory elements in the human genome. Specifically, we investigated whether such enriched regions concurred with existing genome annotations and data for histone modifications. Furthermore, we used the enriched regions to identify chromatin markers that best correlated with the binding of transcription factors and to evaluate previously used markers for regulatory regions. Second, we wanted to investigate whether the combination of transcription factors associated with the enriched regions differed depending on the type of regulatory element to which the enriched region mapped. Specifically, we wondered whether the transcription factor composition differed between enhancers and TSS proximal promoter elements.

Our analysis was based on ChIP-Seq reads for transcription factors from two cell lines: K562 and Gm12878. Totals of 39 and 28 factors, respectively, were mapped in each cell line, and 13 factors were mapped in both cell lines. We used clusters of colocalised transcription factor-binding events as identifiers for regulatory elements involving transcription factors and verified that these clusters generally overlapped with regions of active chromatin. We then used two different strategies to identify four groups of transcription factor clusters with potentially different regulatory roles. First, we examined clusters mapping to previously annotated genes and promoters and separated these into (1) clusters mapping to annotated promoter regions (promoter clusters) and (2) clusters mapping to annotated genes but outside the promoter region (gene clusters). Second, we performed an alternative cluster separation independent of annotations, where clusters mapping to histone modifications closely associated with active transcription (H3K4me3, H3K36me3 and RNA polymerase II (Pol II) binding: transcript clusters) were separated from clusters with potentially distal regulatory function with respect to transcription (enhancer clusters). This definition represents an additional separation of the regulatory elements normally referred to as 'enhancers' [24] into elements that produce transcripts and those that do not. The clusters associated with transcripts also correlated with actual transcription levels from high throughput RNA sequencing (RNA-Seq) data. When comparing clusters in these groups, we observed that the clusters differed in their composition of transcription factors and association with specific histone modifications. Especially, we found that the identified enhancer clusters correlated well with the histone modification H3K4me1, a marker previously used for enhancers, but less well with acetylation of lysine 27 on histone 3 (H3K27ac) or binding of the histone acetyltransferase p300, two other commonly used markers for enhancers. We also investigated whether our selection of transcription factors gave good coverage of all cell type-specific regulatory elements in the human genome and found that a relatively modest selection of factors was sufficient to cover 90% of the annotated promoters for transcribed genes.

## Results

### ChIP-Seq peaks for different transcription factors cluster along the genome

**Figure 1:**
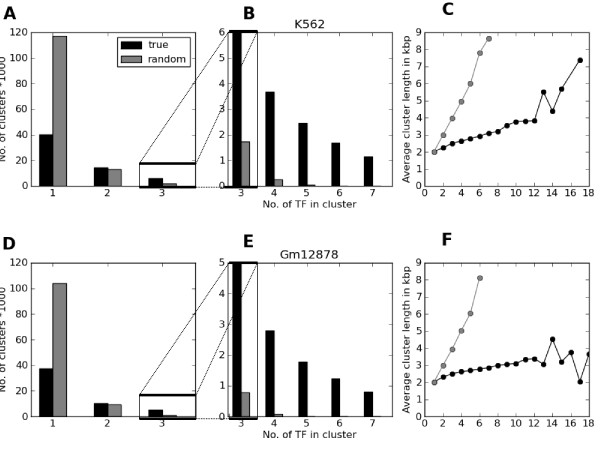
**High-throughput sequencing coupled with chromatin immunoprecipitation (ChIP-Seq) peaks from multiple transcription factors form clusters within the genome**. Number of ChIP-Seq peak clusters compared to the number of clusters produced after a random shuffle of peak positions for K562 **(A) **and **(B) **and Gm12878 **(D) **and **(E)**. The maximum numbers of peaks found in the peak clusters were 17 for K562 and 18 for Gm12878 compared to 7 and 6, respectively, for the random shuffle. **(C) **and **(F) **show average cluster lengths in kilobase pairs with increasing numbers of peaks in the clusters. A smaller increase in peak lengths is observed for the peak clusters, indicating a higher degree of clustering. TF, transcription factor.

Binding sites for transcription factors tend to cluster in regulatory modules [25-27], and recent studies in *Drosophila *[18] and mouse [10,22] have also shown that peaks from a set of transcription factors identified by ChIP-Seq and related methodologies tend to cluster along the genome. To investigate whether human transcription factor ChIP-Seq peaks also display clustering properties, we identified overlaps between ChIP-Seq peaks for 39 factors in K562 and for 28 factors in Gm12878 (see Methods). These overlaps were compared with overlaps obtained by randomly shuffling peak positions within each chromosome. For the 39 factors in K562, a total of 151,624 individual ChIP-Seq peaks were identified. When identifying overlaps, these peaks condensed into 71,311 nonoverlapping regions, where 30,934 regions contained more than one peak. The same procedure for the randomly shuffled peaks produced 132,615 regions, where only 15,373 contained more than one peak. We defined each region containing more than one peak as a transcription factor cluster, and this definition was used throughout the rest of this study. The relative number of real transcription factor clusters compared to random clusters strongly increased with the number of transcription factors mapping to the cluster (Figures 1A and 1B). We also observed an increase in the average length of the random clusters compared to transcription factor clusters as more peaks associated with the cluster (Figure 1C). The latter indicates that peaks within each cluster locate to more similar genomic positions than would be expected by chance. The results were similar for the 28 factors analysed in Gm12878 (Figures 1D and 1F), where 126,238 peaks produced 61,209 nonoverlapping regions, and 23,491 regions contained more than one peak. The corresponding numbers for the random shuffle were 114,157 and 10,148. On the basis of these results, we conclude that human ChIP-Seq peaks from different transcription factors have a strong tendency to form clusters along the genome. We also calculated the overlap between transcription factor clusters in both cell types and found 8,320 overlapping clusters (one-third to one-fourth of all clusters). A comprehensive comparison between cell types will be the topic of a future study (T Håndstad, M B Rye, F Drabløs and P Sætrom, unpublished data). Herein we focus on the cluster patterns within each cell type individually but compare general trends between the cell lines.

### Histone H3 lysine 4 methylation is the chromatin mark best associated with transcription factor binding

**Figure 2:**
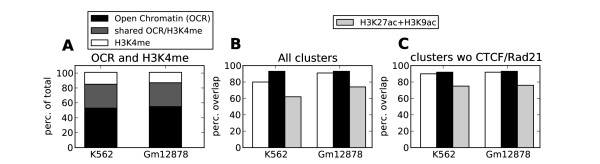
**Methylation of lysine 4 on histone H3 (H3K4me) is a more specific marker of transcription factor clusters than is open chromatin (OCR)**. **(A) **Relative numbers of unique and shared regions for OCR and H3K4me. To compensate for the smaller region lengths in OCR compared to H3K4me, all unique OCR regions within 600 bp of each other were merged. Still, there are about threefold more unique OCR regions (black) than unique H3K4me regions (white). Thus H3K4me is a more precise marker than OCR when comparable fractions of transcription factor (TF) clusters overlap with these regions. **(B) **Percentage of TF clusters that overlap with H3K4me, OCR and combined acetylated lysine 27 on histone 3 (H3K27ac) and H3K9ac. **(C) **Same as graph **(B)**, but with CTCF and Rad21 removed from the clusters. CTCF/Rad21 was the most frequent TF pair encountered in K562. We did not have high-throughput sequencing coupled with chromatin immunoprecipitation data for Rad21 in Gm12878, resulting in more singleton peaks for CTCF in Gm12878, which is the reason for the improved overlap observed in **(B) **for Gm12878.

Presumably, transcription factors require open chromatin for DNA binding, but it is unclear whether open chromatin by itself is a good predictor of transcription factor binding. To address this question, we investigated the overlap between transcription factor clusters and experimentally defined open chromatin regions (OCRs) (see Methods) or regions containing different histone marks associated with open or closed chromatin. Our first observation was the abundance of OCRs compared to regions showing mono-, di- or trimethylation of H3K4, histone modificationstypically associated with accessible chromatin [6,7]. Although the number of regions enriched with H3K4me comprised less than half the number of experimentally defined OCRs (Figure 2A), 54 of 67 transcription factor data sets had overlap with H3K4me similar to or better than that with OCRs (Additional file 1, Figure S1). Exceptions were the pair CTCF/Rad21, which mapped better to OCRs than to H3K4me, and a few other factors, such as NRSF and SETDB1, which mapped to neither H3K4me nor OCRs. When CTCF/Rad21 was excluded, 90% and 92% of the clusters, respectively, mapped to H3K4me compared to 92% and 93% that mapped to OCRs for K562 and Gm12878, respectively (Figures 2B and 2C), despite the much smaller number of H3K4me regions. Only a few clusters (4% in K562 and 3% in Gm12878) mapped to regions without H3K4me, OCRs or H3K27ac and/or H3K9ac. We therefore conclude that H3K4me and OCRs mark somewhat different types of accessible chromatin most visible in the overlap with CTCF/Rad21 elements, but that H3K4me is a more specific signature than OCRs for the binding of most other transcription factors.

**Figure 3:**
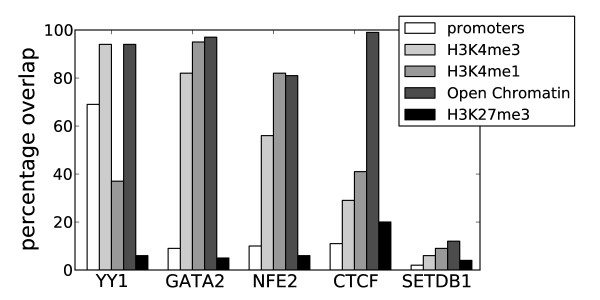
**Examples of transcription factors mapping to different chromatin domains**. YY1 has a preference for annotated promoters compared to the other factors. GATA2 maps to both methylated lysine 4 on histone H3 (H3K4me3) and H3K4me1, but not to annotated promoters, whereas NFE2 has a higher preference for H3K4me1. CTCF maps to open chromatin in addition to H3K4me1 and H3K4me3, whereas SETDB1 does not map well to any domain. As expected, none of the factors mapped well to domains containing the repressive H3K27me3 mark.

Different transcription factors showed a preference for different general chromatin signatures (Figure 3 and Additional file 1, Figure S1). Factors enriched at annotated promoters (for example, YY1) generally mapped to H3K4me3, whereas other factors (for example, GATA2 and NFE2) preferentially mapped to H3K4me1, which may indicate their association with enhancers. Except for a few factors not mapping specifically to any chromatin mark, all factors analysed in this study preferentially mapped to known marks for accessible chromatin. This shows that regions enriched with transcription factors can generally be used as an alternative to histone modifications and other markers (such as DNase hypersensitivity) to identify genomic regions involved in gene regulation. We also found that data for histone modifications associated with accessible chromatin were highly redundant. Regions marked by acetylation (H3K9ac and H3K27ac) were almost totally contained within H3K4me (97% and 98% of combined H3K27ac and H3K9ac overlapped with H3K4me in K562 and Gm12878, respectively), and regions marked by H3K4 mono-, di- and tri-methylation were also highly overlapping (Additional file 1, Figure S2). In addition, H3K27ac and H3K9ac regions where highly overlapping in K562 (83% and 89% overlap, respectively), whereas in Gm12878, H3K9ac regions (92% overlap) covered a subset of H3K27ac regions (69% overlap). Though H3K27ac and H3K9ac correlated well with other marks for accessible chromatin, these regions showed a weaker overlap with clusters compared to H3K4me and OCRs (Figures 2B and 2C). All of these observations taken together show that H3K4me is a good marker for accessible chromatin and seems to be the preferred marker for transcription factor binding.

Few factors were associated with repressive modifications. An important exception is NRSF (also known as REST), which, in contrast to the other modifications we analysed, mainly associated with H3K27me3 (Additional file 1, Figure S1). NRSF is a transcriptional repressor that acts as a scaffold for recruiting several chromatin-modifying complexes involved in dimethylation of H3K9 (H3K9me2) and demethylation of H3K4 [28]. By binding long noncoding RNAs, NRSF can also colocalise with polycomb-repressive complex 2 (PRC2) [29], which may explain the observed association between NRSF and H3K27me3. Similarly, H3K27me3 was partly associated with CTCF/Rad21, which is consistent with CTCF's interacting with PRC2 [30].

### Transcription factor clusters map to promoters of transcribed genes and upstream and/or downstream of promoters of both transcribed and silent genes

**Figure 4:**
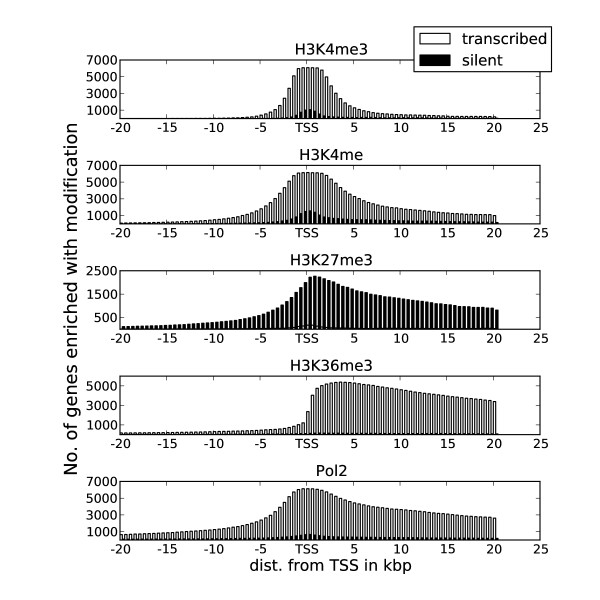
**Histone modifications and RNA polymerase II (Pol II) have distinct profiles around transcription start sites (TSSs) of transcribed and silent genes**. The plots are derived from the K562 cell line. Methylated lysine 4 on histone H3 (H3K4me3) is enriched around TSSs in transcribed genes, H3K36me3 is enriched in gene bodies of transcribed genes and Pol II is enriched at both TSSs and gene bodies of transcribed genes, whereas H3K27me3 is enriched at silent genes. Some H3K4me enrichment was also observed for silent genes. Similar profiles for Gm12878 are shown in Additional file 1, Figure S3.

The binding of transcription factors is closely related to gene transcription. To test whether transcription factor clusters associate with transcribed genes, we investigated the binding pattern of clusters within the set of annotated nonredundant genes (that is, gene clusters; see Methods) and their promoters (that is, promoter clusters) and correlated this with RNA-Seq gene transcription data (see Methods). On the basis of RNA-Seq, we defined 6,228 genes as being highly transcribed in both cell lines and identified 5,591 and 4,124 genes with zero transcription in K562 and Gm12878, respectively. In accordance with previously reported results [7,31,32], H3K4me was enriched around TSSs for the set of transcribed genes, H3K36me3 and Pol II were enriched in their transcribed regions and H3K27me3 was enriched at silent genes (Figure 4 and Additional file 1, Figure S3).

**Figure 5:**
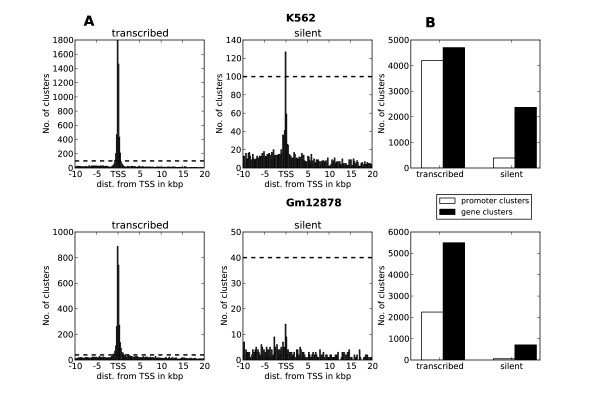
**Transcription factor clusters are strongly enriched around transcription start sites (TSSs) in actively transcribed genes**. **(A) **Mapping of transcription factor clusters around TSSs in annotated genes and promoters for transcribed and silent genes in K562 and Gm12878. Bars correspond to the number of clusters within 200-bp intervals. The dotted line indicates the same level in both graphs and is included to emphasise the difference in axis scales between transcribed and silent genes. **(B) **Percentage of clusters identified as promoter clusters (mapping within 2,000 bp upstream and 200 bp downstream of TSSs) and gene clusters (mapping within 10 kb upstream of TSSs or within the gene body but outside the annotated promoter region). A substantial number of gene clusters mapped to transcribed and silent genes in both cell lines.

In transcribed genes, clusters were highly abundant around TSSs, as expected (Figure 5A). However, we also observed clusters mapping outside the typical promoter region 2 kb upstream and 200 bp downstream from TSSs. The overall number of clusters mapping to the immediate promoter region of transcribed genes (promoter clusters) was comparable to the number of clusters mapping to other parts of the genes (gene clusters), in particular for K562 (Figure 5B). For silent genes, the number of promoter clusters was greatly reduced compared to the number of gene clusters. Thus gene clusters locate to both transcribed and silent genes, whereas promoter clusters mostly locate to transcribed genes. For both transcribed and silent genes, the distribution of clusters showed no clear distance dependence beyond 2 kb from TSSs. This likely reflects promoter clusters' direct regulation of local RNA polymerase recruitment or assembly, whereas gene clusters are involved in long-range regulation.

### Composition of gene clusters correlates with H3K4me and differs from promoter clusters of transcribed genes

**Figure 6:**
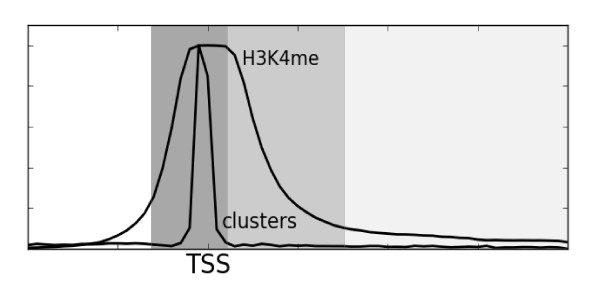
**The genomic region around transcription start sites (TSSs) can be divided into subregions**. This illustration shows promoter cluster locations (dark grey), gene clusters associating with methylated lysine 4 on histone H3 (H3K4me3) (medium grey) and gene clusters not associated with H3K4me (light grey) relative to the TSS. The three cluster types showed clear differences in transcription factor composition.

**Figure 7:**
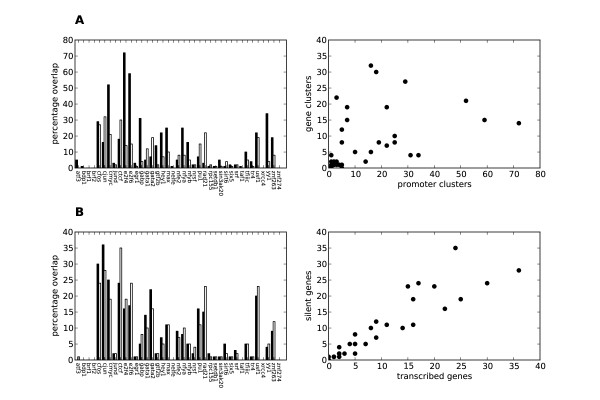
**The composition of transcription factor clusters is correlated between cluster types**. The correlations shown are based on the enrichment of each factor in each cluster type. **(A) **Promoter cluster (black) and gene clusters (white) in transcribed genes (0.46, weak correlation). **(B) **Gene clusters associated with methylated lysine 4 on histone H3 in transcribed (black) and silent (white) genes (0.93, strong correlation). The plots are based on data derived from K562 cells.

To further investigate differences in function between promoter and gene clusters, we asked whether the groups differ in their use of transcription factors. Furthermore, when correlating the locations of transcription factor clusters to histone modifications, we observed that the gene clusters could be further separated into two groups, depending on their association with H3K4me (Figure 6). We therefore used correlation coefficients (r, Pearson's correlation) to measure the difference in transcription factor composition between promoter clusters, gene clusters overlapping with H3K4me and gene clusters not overlapping with H3K4me (Table 1). Specifically, we calculated correlation coefficients by comparing profiles of relative enrichment of transcription factors within each group of clusters (see Methods; see Figure 7 for two examples of profile comparisons).

**Table 1:** Correlation coefficients for composition differences in transcription factor clusters mapping to annotated genes and promoters

Composition correlation	K562	Gm12878
*prom-h - gene-any-h *	0.46	0.04
*prom-z - gene-any-z*	0.75	0.32
*prom-h - prom-z*	0.70	0.37
*gene-any-h - gene-any-z*	0.99	0.99
*gene-any-h - prom-z*	0.75	0.32
*gene-pos-h - gene-neg-h*	0.40	0.56
*gene-pos-z - gene-neg-z *	0.66	0.81
*gene-pos-h - prom-h*	0.52	0.04
*gene-pos-z - prom-z*	0.89	0.39
*gene-pos-h - gene-pos-z*	0.93	0.97
*gene-neg-h - gene-neg-z*	0.99	0.88

*prom*, promoter clusters; *gene-any*, gene clusters; *gene-pos*, gene clusters overlapping with H3K4me; *gene-neg*, gene clusters not overlapping with H3K4me; *h*, genes with high expression; *z*, genes with zero expression.

Consistent with the different regulatory functions of promoter and gene clusters in transcribed genes, these two cluster types showed the largest difference in composition (r 0.46 and 0.04 for K562 and Gm12878, respectively) (see Figure 7A). We also observed a large compositional difference between gene clusters associated with and not associated with H3K4me (r 0.40 and 0.56, respectively) and between gene clusters associated with H3K4me and promoter clusters (r 0.52 and 0.04, respectively) in transcribed genes. Thus transcribed genes had three types of clusters with markedly different composition: promoter clusters close to TSSs, gene clusters more distal to TSSs associated with H3K4me, and gene clusters far into the gene body not associated with H3K4me.

When comparing clusters between transcribed and silent genes, we observed that the composition of gene clusters did not change with respect to transcription (r 0.99 and 0.99, respectively) and that this was true both for gene clusters associated with H3K4me (r 0.93 and 0.97, respectively) (Figure 7B) and for those not associated with H3K4me (r 0.99 and 0.88, respectively). In contrast, and consistent with promoter clusters' having active and local roles in transcription regulation, promoter clusters changed in composition between transcribed and silent genes (r 0.70/0.37, respectively). Thus there is a change in composition between transcribed and silent genes only for promoter clusters, whereas the composition in gene clusters remains similar. This indicates that long-range regulatory interactions are present in both transcribed and silent genes. Further supporting this conclusion, CTCF, which facilitates long-range interactions [3], was among the most abundant transcription factors in all regions except for promoters of transcribed genes (Additional file 1, Table S4).

For silent genes, the compositional differences between the three types of clusters showed some discrepancies between the two cell lines. Especially for K562, the composition of gene clusters associated with H3K4me mostly resembled the composition of promoter clusters (r 0.89), whereas the closest resemblance was observed with gene clusters not associated with H3K4me in Gm12878 (r 0.81) (see Discussion).

The enrichment of individual transcription factors in promoters and genes did not always reflect the composition of the different cluster groups. Most factors were present in several groups, but the relative enrichment of each factor in each group was sometimes very different (Figure 7 and Additional file 1, Table S4). For example, in transcribed genes, a higher number of promoter clusters contained the factor c-Jun (831) compared to gene clusters (583), but the percentage of clusters containing c-Jun was slightly higher in gene clusters (24%) than in promoter clusters (16%). Thus c-Jun may have a more important regulatory role in gene clusters, even though more ChIP-Seq peaks for this factor mapped to promoter clusters than to gene clusters. Generally, the 11,041 c-Jun peaks mapped better to TSS distal markers, such as H3K4me1 (Additional file 1, Figure S1a) than to promoters, which indicates the involvement of c-Jun in long-range regulatory interactions.

We also noted some H3K4me and Pol II enrichment at promoters of silent genes. However, this enrichment was not transformed into transcriptional output, as evidenced by the zero RNA-Seq expression and low enrichment of H3K36me3 for these genes (Figure 4 and Additional file 1, Figure S3). The increased enrichment of these transcript-related features may explain the higher concentration of transcription factor clusters around TSSs for silent genes in K562 compared to Gm12878 (Figure 5A).

### H3K4me3, H3K36me3 and Pol II identify clusters overlapping with transcripts

In addition to annotated promoters and genes, we expected a proportion of the transcription factor clusters to associate with transcripts and enhancers outside annotated regions. To separate clusters located to promoters or to the body of transcripts (transcript clusters, including possibly unannotated transcripts) from clusters not associated with promoters or transcript bodies (enhancer clusters), we used associations with histone modifications H3K36me3 and H3K4me3, together with enrichment of Pol II, as described by Mikkelsen *et al*. [31]. Because of the special regulatory function of CTCF/Rad21, these two factors were left out of the analysis at this stage. Clusters overlapping with either H3K36me3 or Pol II were classified as transcript clusters. In addition, clusters overlapping with H3K4me3 were classified as transcript clusters if the region of H3K4me3 enrichment overlapped with either H3K36me3 or Pol II. The last criterion separated isolated regions of H3K4me3 from regions of H3K4me3 that involved the other two transcription markers. We used independent data for Pol III to identify additional transcript clusters not transcribed by Pol II.

Three points must be mentioned with respect to the classification of transcript and enhancer clusters. First, we have used the term 'enhancer clusters' to describe clusters which do not contain the histone modifications and polymerase signatures characteristic of transcription and have indicated that these are more likely to be involved in long-range interactions. However, a recent study [33] showed that a subset of enhancers involved in long-range interactions also produce short noncoding transcripts. Since such enhancers also recruit Pol II and show enrichment of H3K36me3 [34], these regulatory elements are classified among the transcript clusters according to the definition given above. Second, a subset of enhancer clusters may represent elements that are not involved in direct gene regulation [35,36]. Third, when we compared this data-driven classification with our previous annotation-based analysis, we observed some transcript clusters in promoters and gene bodies of silent genes, especially in K562. The set of silent genes showed a small enrichment of Pol II and H3K4me3 (but not H3K36me3) around their TSSs (Figure 4 and Additional file 1, Figure S3), but this enrichment did not result in detectable transcription. We still chose to classify clusters in these silent gene regions as transcript clusters, as these signatures were most likely a result of stalled transcription [37] and not related to long-range interactions. So, though there may be different and possibly overlapping functions between the two classes of regulatory elements, we continue to use the notion of transcript clusters as mainly transcript-producing and enhancer clusters as mainly involved in long-range interactions throughout the rest of the text.

**Figure 8:**
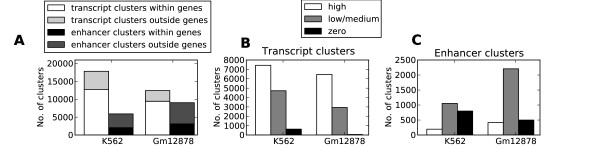
**Transcript and enhancer clusters mapping within and outside annotated genes and correlation with actual transcripts**. **(A) **Number of transcript and enhancer clusters mapping within and outside annotated genes and promoters in both cell lines. **(B) **Number of transcript clusters mapping to genes with high, low/medium and zero expression in both cell lines as measured by high throughput RNA-sequencing (RNA-seq). **(C) **Number of enhancer clusters mapping to genes with high, low/medium and zero expression in both cell lines as measured by RNA-Seq. **Methylation of lysine 4 on histone H3 (H3K4me) is a more specific marker of transcription factor clusters than is open chromatin (OCR)**

In K562 and Gm12878, we identified 17,836 and 12,424 transcript clusters, respectively, and 5,914 and 9,056 enhancer clusters, respectively. As expected, most of the transcript clusters and a minority of the enhancer clusters in K562 and Gm12878 mapped to annotated promoters and genes (12,775 and 9,458 transcript clusters, respectively, and 2,044 and 3,118 enhancer clusters, respectively) (Figure 8A). To validate these transcript clusters, we investigated their overlap with genes with respect to high, medium/low and zero gene expression as measured by RNA-Seq (Figures 8B and 8C). Over 95% of the transcript clusters in K562 and 99% in Gm12878 mapped to genes with high or medium/low expression. The enhancer clusters were more evenly distributed among the three expression states, with a slight bias towards genes with medium/low or zero expression. Both results confirmed that the model based on H3K36me3, H3K4me3 and Pol II could separate enhancer clusters from transcript clusters associated with annotated genes and promoters.

We primarily used Pol II-related transcription to separate transcript clusters from enhancer clusters. Transcripts can be produced by polymerases other than Pol II, and clusters associating with these polymerases should be identified by our model as long as they also associate with H3K36me3 and H3K4me3. However, we did not always observe this association when analysing independent data for Pol III. The overlap between Pol III and H3K36me3 was only 39% and 26%, compared to 72% and 71% for Pol II and H3K36me3, in K562 and Gm12878, respectively. We therefore included the independent Pol III data in our model. On the one hand, we do not know whether other polymerases behave similarly to Pol II or Pol III with respect to H3K36me3 and H3K4me3, so we cannot exclude the possibility that some clusters classified as enhancer clusters may be associated with transcription by other polymerases, such as Pol I. The effect of Pol I transcription may, on the other hand, be most pronounced in genomic repeat regions, which are often excluded when mapping ChIP-Seq data to the genome.

A large number of clusters mapped outside annotated genes and promoters (5,061 and 2,966 transcript clusters and 3,870 and 5,938 enhancer clusters in K562 and Gm12878, respectively) (Figure 8A). To validate transcript clusters mapping outside annotated genes and promoters, we used data from two independent studies that identified promoters for miRNA transcripts [38] and long, intergenic, noncoding RNA (lincRNA) transcripts [39]. The miRNA TSSs were cell type-independent, whereas the lincRNAs were mapped in another cell line. We found an increased overlap between these noncoding RNA transcripts and our transcript clusters compared to enhancer clusters and overlaps expected by chance (Table 2). Though the independent data covered only a small part of the transcript clusters outside annotated genes and promoters, the good correspondence between transcript clusters and data from RNA-Seq, miRNA and lincRNA lead us to conclude that the transcript clusters did represent transcript-related regulatory elements. Conversely, we concluded that the enhancer clusters were enriched with long-range regulatory elements.

**Table 2:** Overlap between independent miRNA and lincRNA transcripts and transcript and enhancer clusters outside annotated genes and promoters

Cell type	Transcript	Number of transcripts	Average length	**Overlap with transcript clusters**^**a**^	**Overlap with enhancer clusters**^**a**^
K562	miRNA	227	862	27 (2)	4 (1)
Gm12878	miRNA	230	853	18 (1)	2 (2)
K562	lincRNA	592	24,502	97 (22)	29 (18)
Gm12878	lincRNA	586	24,536	53 (11)	33 (26)

^a^Number of overlaps expected by chance are shown in parentheses.

### H3K4me1 is a better marker for enhancer clusters than p300 or H3K27ac

**Figure 9:**
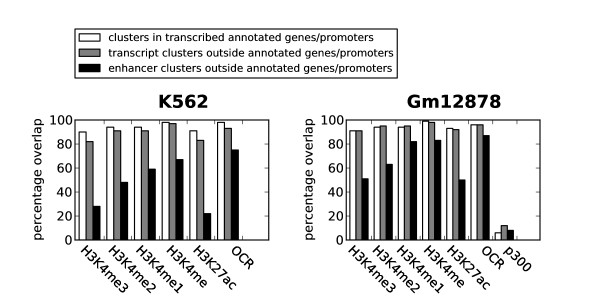
**Transcript and enhancer clusters show different overlap with histone modifications and open chromatin (OCR)**. Transcript clusters generally show a good overlap with all modification marks for accessible chromatin together with OCR, whereas enhancer clusters have a preference for methylated lysine 4 on histone H3 (H3K4me1) over H3K4me3 and acetylated lysine 27 on histone 3 (H3K27ac). The acetyltransferase p300 did not show a strong preference for enhancer clusters in Gm12878.

Though attempts have been made to annotate enhancers [20,21,40-42], the general impression is that enhancers remain poorly annotated in the human genome. The most likely transcription factor clusters associated with enhancers are clusters that lack the histone modification marks characteristic of transcription (H3K4me3, H3K36me3 and Pol II; see previous section). We therefore wondered whether these clusters, which we termed 'enhancer clusters', showed marks previously associated with enhancer regions. We focused our analysis on enhancer clusters mapping outside annotated genes and promoters. There were 3,870 clusters of this type in K562 and 5,938 in Gm12878. We then investigated the overlap of these clusters with respect to three recently used marks for enhancers: histone modifications H3K4me1 and H3K27ac and binding of the histone acetyltransferase p300 (Figure 9 and Additional file 1, Figure S5). Several studies have suggested that H3K4me1 is an enhancer marker [16,20,21,32,43], and we also observed an enrichment of H3K4me1 relative to other histone modifications within our enhancer clusters. Our results thus confirm H3K4me1 as a good marker for enhancers in both cell lines.

Another marker commonly used for enhancer identification is the transcription factor and histone acetyltransferase p300 [16,20,21,40,43,44]. However, we did not observe a good correspondence between this factor and enhancer clusters in Gm12878. Only 8% of the enhancer-related clusters were covered by this factor (data for p300 in K562 were not available). In fact, we identified ten factors in Gm12878 with better coverage of the enhancer clusters than p300, the best ones being BATF (51%), IRF4 (45%) and PU1 (41%). The latter factors also showed a preference for H3K4me1 (Additional file 1, Figure S1B), whereas p300 also mapped well to H3K4me3 and transcript clusters.

Although we saw poor correspondence between p300 and regulatory elements in Gm12878, p300 could be a better marker in other cell lines [40]. Only approximately 1,500 p300 peaks were identified by the ChIP-Seq analysis, which is one reason for the low coverage in Gm12878. In addition, the identified peaks did not seem to map specifically to enhancers. We did not observe any transcription factors with specificity only towards the enhancer related clusters, and all factors with a high overlap with these clusters also overlapped well with transcript clusters (Additional file 1, Figure S5).

H3K27ac has also recently been used as an identifier for enhancer regions [36,45], but it showed a weaker overlap with enhancer clusters compared to H3K4me1 (Figure 9). In addition, all enhancer clusters containing H3K27ac also contained H3K4me1. Still, H3K27ac has been shown to be a useful marker for separating active from weak enhancers [34,36] and might provide valuable information on subclasses of enhancers and enhancer activity in addition to the H3K4me1 mark. The notion that H3K27ac marks specifically active regulatory elements is reinforced by the observation that H3K27ac is present at nearly all active genes (Figure 9). We also observed that H3K9ac shows a mapping pattern similar to that of H3K27ac, especially in K562 (not shown). The percentage overlap of H3K27ac was also similar to that of H3K4me3 (Figure 9); however, these two modifications mark somewhat different enhancer clusters. OCRs also showed good overlap with enhancer clusters, but an OCR in itself was a less specific marker for enhancer clusters because of the large number of OCRs not mapping to any transcription factors (see Discussion). Thus the overall conclusion is that, in our enhancer clusters bound by transcription factors, H3K4me1 was a superior individual marker compared to H3K27ac, p300 and OCRs.

### Mapped transcription factors cover a high percentage of promoters in highly transcribed genes

**Figure 10:**
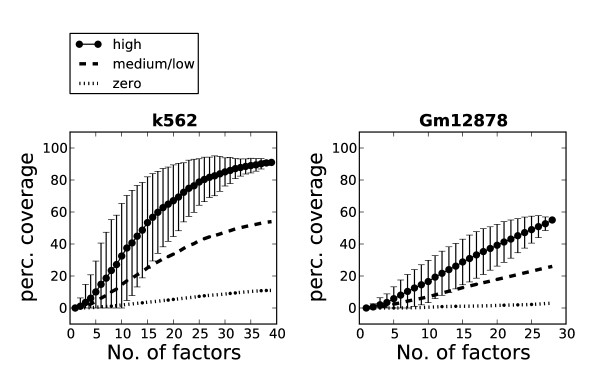
**Transcription factors mapped by high-throughput sequencing coupled with chromatin immunoprecipitation (ChIP-Seq) show good coverage of annotated promoters for transcribed genes**. Graphs showing the percentage of annotated gene promoters covered by clusters in K562 and Gm12878. The genes are divided into groups with high, medium/low and zero expression.

There are 1,500 to 2,000 known transcription factors binding to DNA in humans [46,47]. Because not all factors are expressed in each cell type and since factors generally bind to DNA in a combinatorial fashion, we investigated whether our selected clusters were sufficient to cover regulatory regions in a specific cell type. We investigated the ability to cover annotated promoters for genes with high, medium/low and zero expression in both cell lines (Figure 10). The coverage estimate was based on an average of 100 calculations whereby the order in which the transcription factors were selected was randomly shuffled between each calculation. As more transcription factors are added, the percentage of regulatory elements covered by clusters increases. For promoters of highly expressed genes, the coverage reaches 91% for K562 and 55% for Gm12878 when all transcription factors are added. The coverage was lower in promoters of genes with medium/low and zero expression, which is consistent with these genes' having low or no transcription in the two cell types. To investigate whether the inclusion of singletons would increase the coverage further, we repeated the analysis, but now also including singleton peaks in addition to clusters (Additional file 1, Figure S6). When singletons were included, the coverage increased to 96% for K562 and 80% for Gm12878. An interesting observation regarding K562 is that when the number of factors exceeds 30, the increase in coverage of highly transcribed genes by adding a new factor is only marginal. This trend was also observed when we investigated coverage of the identified transcript and enhancer clusters by the same procedure (Additional file 1, Figure S6). Thus, despite the large number of existing human transcription factors, the results derived from the coverage analysis indicate that a relatively modest number of factors may be sufficient to map regulatory elements in a specific cell line.

## Discussion

### Observed cluster differences are not due to noisy ChIP-Seq peaks

ChIP-Seq data are potentially noisy [48-50], so it was important to separate noisy peaks from true binding events in our study. Given the clustering properties of transcription factors, we regarded overlapping peaks as more confident than singleton peaks, which led us to focus on peak clusters rather than individual peaks when defining regulatory transcription factor elements. We validated this approach (that is, using peaks in clusters) by sorting peaks for each of the 67 transcription factor data sets into 20 bins according to increasing ChIP-Seq tag intensity so that the most intense peaks were associated with the highest bin number. The largest number of singleton peaks was found in bin 3, whereas the largest number of peaks in clusters of size both 2 and 3 were found in bin 11. This indicates that a higher fraction of the singleton peaks are potentially noisy and no gain in peak confidence is realised by increasing the cluster size limit from 2 to 3. We thus decided to use a cluster size limit of 2 in our study. To further verify that peaks in genes clusters were not due to noise, we investigated the confidence of peaks in gene clusters versus promoter clusters. Though we observed a higher average cluster size in promoter clusters than in gene clusters (Additional file 1, Table S4), only minor differences in the average bin value were observed, meaning that ChIP-Seq peaks in gene clusters are not more likely to be noise than peaks in promoter clusters. The reason for the decreased average size of gene clusters could be that fewer factors in these clusters were mapped by ChIP-Seq or that these clusters generally contain fewer transcription factors. We also investigated whether the difference in transcription factor composition among the cluster groups shown in Table 1 changed when we increased the cluster size limit to 3 and 4. Though we generally observed an increase in the correlation values with increasing cluster size limit, the relative differences between the groups stayed the same (data not shown).

Not all singleton peaks are noise. In fact, some of the singleton peaks are among the high-confidence peaks within their data sets. However, by focusing on peak-overlaps, we could concentrate our analysis on regulatory regions containing several peaks, even if each peak in the region was less confident when evaluated on its own. We also considered the effect of losing a few singleton peaks and candidate regulatory regions preferable to including false-positive singleton peaks, which would lead to many additional false regulatory elements.

### Clusters and chromatin signatures show discrepancies between the two cell lines

When mapping transcription factor clusters to annotated genes, our results agreed with the common notion that transcription factors in promoter regions drive the recruitment of the transcription initiation complex leading to gene transcription. Clusters were highly enriched within the typical promoter region (-2,000 bp to +200 bp) of the TSSs in transcribed genes and depleted in silent genes. We also identified a significant number of clusters within genes, which, compared to promoter clusters, showed little change in abundance and composition between transcribed and silent genes. Several studies have reported transcription factor binding outside the typical promoter region [51,52], but the extent and biological significance of such binding events have generally been studied less often. Some of the regulatory roles of transcription factors in these TSS distal clusters could be to modulate the chromatin environment around genes, to work as enhancer elements directed towards their own gene or distant genes, or to regulate individual transcription of noncoding RNA within the gene body. The correlation in composition between some of these clusters and H3K4me should indicate a role in chromatin modulation. The total number of gene clusters was higher in transcribed genes than in silent genes (Figure 5B). Studies of three-dimensional chromosome organisation inside the nucleus [53] have revealed that accessible and closed chromatin tend to compartmentalise, with the consequence that transcribed genes associated with accessible chromatin have a higher probability of being spatially close to other transcribed genes than to silent genes associated with closed chromatin. A high degree of dynamic intra- and interchromosomal interactions are often observed in the accessible compartment, which may explain the higher frequency of gene clusters in transcribed genes than in silent genes. Our observation that CTCF was one of the factors most enriched in gene clusters (Figure 7 and Additional file 1, Table S4) may also point in this direction, since CTCF is involved in higher-order organisation and modulation of chromatin domains [3,54,55].

The discrepancies observed when we compared cluster composition in the two cell lines may have several explanations. The difference in similarity between promoter clusters in transcribed and silent genes for the two cell lines (r 0.70 in K562 vs 0.37 in Gm12878) may partly be explained by the somewhat higher enrichment of Pol II at silent promoters in K562 (compare Figure 4 with Additional file 1, Figure S3). When we removed Pol II-associated promoter clusters from silent genes in K562, the correlation dropped from 0.70 to 0.42, which is more similar to Gm12878. It is thus likely that promoter clusters at silent genes in K562 are more enriched in factors directly involved in transcription, leading to the increased composition similarity with promoter clusters of transcribed genes. Additional observations indicate that transcription factors mapped in K562 are generally more promoter-specific and directly related to transcription than the factors mapped in Gm12878. First, the number of gene clusters relative to promoter clusters is higher in Gm12878 than in K562 (Figure 5 and Additional file 1, Table S4). Second, when we investigated the transcription factor composition differences between enhancer clusters and clusters mapping to annotated promoters in transcribed genes, we observed a larger difference for K562 (r 0.65) than for Gm12878 (r 0.91). Third, the higher influence of singletons for Gm12878 in the coverage analysis also indicates that Gm12878 promoters are less mapped by transcription factors than promoters in K562. The latter difference could also be reinforced by the smaller number of factors mapped in Gm12878 (28) compared to K562 (39).

### More transcript clusters mapped to silent genes in K562 (625) compared to Gm12878 (56)

Further examination of the 625 transcript clusters mapping to silent genes in K562 revealed that 100 clusters contained H3K36me3, which may indicate transcriptional events not captured by RNA-Seq. Another 188 clusters contained the silent histone modification H3K27me3 in addition to the active marks, which relates these clusters to bivalent domains [37,56]. In promoters of bivalent genes the transcriptional machinery, including Pol II, is recruited, but transcription elongation is stalled and no transcriptional output is produced. We could not find the exact cause for the association of the other 337 transcript clusters to silent genes. Another source of the cell type-specific discrepancies is the selection of transcription factors mapped by ChIP-Seq in each cell line. Of the 39 factors mapped in K562 and the 28 mapped in Gm12878, only 13 factors were common to both cell lines. The selected transcription factors can thus be biased towards specific types of clusters, leading to inconsistent results when the composition profiles are compared. We cannot rule out effects of possible cell type-specific regulation mechanisms, which have been observed in recent studies [43,57].

### Many regions enriched with H3K4me and open chromatin regions are not mapped by any transcription factors

**Figure 11:**
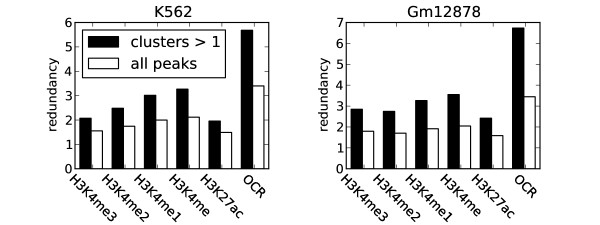
**The total number of regions with histone modifications and open chromatin (OCR) are redundant compared to the number of regions mapped by transcription factors**. The redundancy is calculated as the ratio of total regions enriched with the histone modification to the same regions occupied by a transcription factor cluster. Results are shown both with (all peaks) and without (clusters > 1) singletons. H3K4me1, methylated lysine 4 on histone H3; H3K27ac, acetylated lysine 27 on histone 3.

We generally observed that clusters associated with both H3K4me and OCRs. However, large parts of the genome are enriched with H3K4me and OCRs without being associated with clusters (Figure 11). H3K4me1, for example, is enriched in about three times as many regions as those mapped by clusters, whereas the corresponding overrepresentation of OCRs regions is about six times. When singletons are included, the corresponding enrichment ratios are about 2 and 3.5 for H3K4me1 and OCRs, respectively. If H3K4me1 is regarded as a good marker for enhancers, there is a discrepancy between the coverage of clusters observed at annotated promoters (90% and 70% in K562 and Gm12878, respectively) compared to the cluster coverage of regions enriched with H3K4me1 (50%). If the 90% and 70% (in K562 and Gm12878, respectively) coverage of clusters observed in promoters of active genes is representative of the coverage of regulatory elements in general, one would expect the percentage of H3K4me1 regions containing transcription factors to be much larger than the observed 50%. One reason for this discrepancy could be that the mapping of enhancer-binding transcription factors is sparse compared to those binding to promoters, leading to less coverage of enhancers. Another possible explanation is that histone signatures such as H3K4me1 may be involved in processes other than facilitating transcription factor binding. The larger excess of OCRs might be due to the specificity of the cutting enzyme in DNase hypersensitivity [58] or FAIRE-Seq [59], which also may cut chromatin in regions where transcription factors are not likely to bind. The large number of regions identified by OCRs, as well as the observation that several of these regions are not mapped by any transcription factors, increases the possibility that many OCRs are not functional regulatory elements. Though regions containing H3K4me1 and OCRs are good candidates for enhancers, the additional binding of certain transcription factors to these regions are generally considered a reinforcement of its actual enhancer functionality [24]. In addition, several of the enhancer clusters mapped to none of the signatures of accessible chromatin (25% in K562 and 13% in Gm12878). This observation indicates that not all of the enhancer-related clusters are associated with active gene regulation, but may be involved in other functions and associated with other chromatin marks not mapped in this study.

## Conclusion

In this study, we investigated transcription factor clusters and their relation to gene annotations and chromatin environments throughout the human genome. Our results provide new insight into the relationship between transcription factor binding in regulatory regions and histone modification domains. Specifically, we found that transcription factor clusters mapping to genes outside the core promoter showed similar composition in both transcribed and silent genes, but that the composition differed depending on the presence of H3K4me. H3K4me was also identified as the preferred mark for transcription factor binding in general and should thus be used as a priority marker for identifying transcription factor-binding events in as yet uncharacterised cell types. We also confirmed that the histone modifications H3K36me3 and H3K4me3, together with Pol II, could be used to separate clusters involved in transcription from clusters more related to long-range interactions, although the functions of these two classes of clusters may overlap somewhat. For the latter clusters, H3K4me1 was identified as the preferred marker compared to H3K27ac, p300 and OCRs. The further integration of high-throughput sequencing data of new histone modifications, transcription factors and other regulatory features in different cell types will most certainly increase our knowledge of the complex relationships between transcription factors and histone modifications.

## Methods

### Downloaded data

In this study, we used data from the ENCODE project contained in the UCSC Genome Browser Database [60] and downloaded ChIP-Seq data for the two cell lines K562 and Gm12878. The data consisted of ChIP-Seq reads for 67 transcription factors (26 unique in K562, 15 unique in Gm12878 and 13 mapped in both cell lines) and 9 histone modifications mapped in both cell lines. In addition, we downloaded accessible chromatin tracks analysed by DNase hypersensitivity and FAIRE-Seq (OCR), expression data analysed by RNA-Seq for the same cell lines and gene annotations from UCSC refGene. An overview of all data downloaded from ENCODE database in the UCSC Genome Browser is given in Additional file 1, Table S7. In addition, we downloaded transcript data for miRNA [38] and lincRNA (lincRNA pipeline based on the paper by Guttman *et al*. [39]) from other sources to evaluate cluster classifications outside annotated genes and promoters. The liftOver tool was used to convert the hg19 to the hg18 version of the human genome assembly on both of these data sets using the Galaxy web-based platform (http://main.g2.bx.psu.edu/) for miRNA and the UCSC Genome Browser Database for lincRNA.

### Identification of clusters

Confident ChIP-Seq peaks for each transcription factor were identified by using an in-house method [48] based on output from the programs MACS [61] and SISSRs [62]. To make sure that peaks belonging to the same regulatory element were identified in the same cluster, we extended each peak to 2,000 bp to emulate the common standard used for promoter regulatory elements (2,000 bp upstream and 200 bp downstream from TSSs). Peaks overlapping within the 2,000 bp extension were identified as belonging to the same cluster.

### Identification of random clusters

Within each chromosome, all peak starts were randomly shuffled. Because the full chromosome cannot be mapped by ChIP-Seq, the length of the chromosome was adjusted by the mappability factor in MACS (0.88 for 25-bp tag lengths). Each peak was then extended to 2,000 bp, and overlaps were identified by using the same procedure as that used for the true peaks.

### Identification of domains of histone modifications

ChIP-Seq data for histone modification data and Pol II were analysed by using the program SICER version 1.03 [63]. The gap size parameters were set to 200 for H3K4me3 and to 600 for other histone modifications as recommended [63]. For Pol II, we used a larger gap size of 1,000 to capture longer domains of Pol II binding rather than local Pol II peaks. ChIP-Seq data sets for the same modification were combined, resulting in a single track for each modification in each cell line. To account for the difference in domain size of histone modifications, we split each domain into regions with a maximum length of 5,000 bp and used these regions throughout most of the study. The full-length regions were used only during the identification of transcript clusters by H3K36me3, H3K4me3 and Pol II.

### Gene expression and promoters

Gene expression was measured by the average RNA-Seq intensity based on tags mapped to exons for each gene. Nonredundant genes were selected by grouping all genes with overlapping subsets of exons transcribed from the same strand, then only the gene with the highest expression was selected from each group. From among the downloaded list of 30,399 genes, 18,682 nonredundant genes were identified by using this approach. A set of transcribed genes was chosen as the top third of all redundant genes when sorted by expression level, whereas a set of silent genes was chosen as all genes with zero expression. This resulted in 6,228 transcribed genes (both cell lines) and 5,591 and 4,124 silent genes in K562 and Gm12878, respectively. Promoters were defined as extending 2,000 bp upstream and 200 bp downstream of TSSs.

### Composition correlations

Correlation of transcription factor composition between two types of clusters was calculated in the following way. For both cluster types, the percentage of clusters occupied by each factor was calculated, giving a vector of enrichment for each factor in each cluster type. The Pearson's r correlation between the vector for each cluster type was then calculated by using the corrcoef function in Numpy (http://numpy.scipy.org/).

## Abbreviations

miRNA: microRNA; OCR: open chromatin region; TSS: transcription start site; ChIP-Seq: chromatin immunoprecipitation coupled with high-throughput sequencing.

## Authors' contributions

MBR, PS, FD and TH conceived the study, and MBR designed the study. MBR analysed the data. TH and PS contributed to data analysis. MBR, PS and FD wrote the manuscript. All authors read and approved the final manuscript.

## Supplementary Material

Additional file 1 **Supplementary figures and Tables S1 to S7**. Click here for file

## References

[B1] Farnham PJ Insights from genomic profiling of transcription factors Nat Rev Genet 2009 10 605 616 10.1038/nrg2636 19668247 PMC2846386

[B2] Kagey MH Newman JJ Bilodeau S Zhan Y Orlando DA van Berkum NL Ebmeier CC Goossens J Rahl PB Levine SS Taatjes DJ Dekker J Young RA Mediator and cohesin connect gene expression and chromatin architecture Nature 2010 467 430 435 10.1038/nature09380 20720539 PMC2953795

[B3] Phillips JE Corces VG CTCF: master weaver of the genome Cell 2009 137 1194 1211 10.1016/j.cell.2009.06.001 19563753 PMC3040116

[B4] Kouzarides T Chromatin modifications and their function Cell 2007 128 693 705 10.1016/j.cell.2007.02.005 17320507

[B5] Li B Carey M Workman JL The role of chromatin during transcription Cell 2007 128 707 719 10.1016/j.cell.2007.01.015 17320508

[B6] Bernstein BE Meissner A Lander ES The mammalian epigenome Cell 2007 128 669 681 10.1016/j.cell.2007.01.033 17320505

[B7] Barski A Cuddapah S Cui K Roh TY Schones DE Wang Z Wei G Chepelev I Zhao K High-resolution profiling of histone methylations in the human genome Cell 2007 129 823 837 10.1016/j.cell.2007.05.009 17512414

[B8] Johnson DS Mortazavi A Myers RM Wold B Genome-wide mapping of *in vivo *protein-DNA interactions Science 2007 316 1497 1502 10.1126/science.1141319 17540862

[B9] Park PJ ChIP-seq: advantages and challenges of a maturing technology Nat Rev Genet 2009 10 669 680 19736561 10.1038/nrg2641 PMC3191340

[B10] Chen X Xu H Yuan P Fang F Huss M Vega VB Wong E Orlov YL Zhang W Jiang J Loh YH Yeo HC Yeo ZX Narang V Govindarajan KR Leong B Shahab A Ruan Y Bourque G Sung WK Clarke ND Wei CL Ng HH Integration of external signaling pathways with the core transcriptional network in embryonic stem cells Cell 2008 133 1106 1117 10.1016/j.cell.2008.04.043 18555785

[B11] Niu W Lu ZJ Zhong M Sarov M Murray JI Brdlik CM Janette J Chen C Alves P Preston E Slightham C Jiang L Hyman AA Kim SK Waterston RH Gerstein M Snyder M Reinke V Diverse transcription factor binding features revealed by genome-wide ChIP-seq in *C. elegans* Genome Res 2011 21 245 254 10.1101/gr.114587.110 21177963 PMC3032928

[B12] Hon G Wang W Ren B Discovery and annotation of functional chromatin signatures in the human genome PLoS Comput Biol 2009 5 e1000566 10.1371/journal.pcbi.1000566 19918365 PMC2775352

[B13] Wang Z Zang C Rosenfeld JA Schones DE Barski A Cuddapah S Cui K Roh TY Peng W Zhang MQ Zhao K Combinatorial patterns of histone acetylations and methylations in the human genome Nat Genet 2008 40 897 903 10.1038/ng.154 18552846 PMC2769248

[B14] Ernst J Kellis M Discovery and characterization of chromatin states for systematic annotation of the human genome Nat Biotechnol 2010 28 817 825 10.1038/nbt.1662 20657582 PMC2919626

[B15] Hon G Ren B Wang W ChromaSig: a probabilistic approach to finding common chromatin signatures in the human genome PLoS Comput Biol 2008 4 e1000201 10.1371/journal.pcbi.1000201 18927605 PMC2556089

[B16] Heintzman ND Stuart RK Hon G Fu Y Ching CW Hawkins RD Barrera LO Van Calcar S Qu C Ching KA Wang W Weng Z Green RD Crawford GE Ren B Distinct and predictive chromatin signatures of transcriptional promoters and enhancers in the human genome Nat Genet 2007 39 311 318 10.1038/ng1966 17277777

[B17] Yu H Zhu S Zhou B Xue H Han JD Inferring causal relationships among different histone modifications and gene expression Genome Res 2008 18 1314 1324 10.1101/gr.073080.107 18562678 PMC2493438

[B18] modENCODE Consortium Roy S Ernst J Kharchenko PV Kheradpour P Negre N Eaton ML Landolin JM Bristow CA Ma L Lin MF Washietl S Arshinoff BI Ay F Meyer PE Robine N Washington NL Di Stefano L Berezikov E Brown CD Candeias R Carlson JW Carr A Jungreis I Marbach D Sealfon R Tolstorukov MY Will S Alekseyenko AA Artieri C Booth BW Identification of functional elements and regulatory circuits by *Drosophila *modENCODE Science 2010 330 1787 1797 21177974 10.1126/science.1198374 PMC3192495

[B19] Kharchenko PV Alekseyenko AA Schwartz YB Minoda A Riddle NC Ernst J Sabo PJ Larschan E Gorchakov AA Gu T Linder-Basso D Plachetka A Shanower G Tolstorukov MY Luquette LJ Xi R Jung YL Park RW Bishop EP Canfield TK Sandstrom R Thurman RE MacAlpine DM Stamatoyannopoulos JA Kellis M Elgin SC Kuroda MI Pirrotta V Karpen GH Park PJ Comprehensive analysis of the chromatin landscape in *Drosophila melanogaster* Nature 2011 471 480 485 10.1038/nature09725 21179089 PMC3109908

[B20] Won KJ Chepelev I Ren B Wang W Prediction of regulatory elements in mammalian genomes using chromatin signatures BMC Bioinformatics 2008 9 547 10.1186/1471-2105-9-547 19094206 PMC2657164

[B21] Won KJ Ren B Wang W Genome-wide prediction of transcription factor binding sites using an integrated model Genome Biol 2010 11 R7 10.1186/gb-2010-11-1-r7 20096096 PMC2847719

[B22] Kim J Chu J Shen X Wang J Orkin SH An extended transcriptional network for pluripotency of embryonic stem cells Cell 2008 132 1049 1061 10.1016/j.cell.2008.02.039 18358816 PMC3837340

[B23] ENCODE Project Consortium Birney E Stamatoyannopoulos JA Dutta A Guigó R Gingeras TR Margulies EH Weng Z Snyder M Dermitzakis ET Thurman RE Kuehn MS Taylor CM Neph S Koch CM Asthana S Malhotra A Adzhubei I Greenbaum JA Andrews RM Flicek P Boyle PJ Cao H Carter NP Clelland GK Davis S Day N Dhami P Dillon SC Dorschner MO Fiegler H Identification and analysis of functional elements in 1% of the human genome by the ENCODE pilot project Nature 2007 447 799 816 10.1038/nature05874 17571346 PMC2212820

[B24] Bulger M Groudine M Functional and mechanistic diversity of distal transcription enhancers Cell 2011 144 327 339 10.1016/j.cell.2011.01.024 21295696 PMC3742076

[B25] Blanchette M Bataille AR Chen X Poitras C Laganière J Lefèbvre C Deblois G Giguère V Ferretti V Bergeron D Coulombe B Robert F Genome-wide computational prediction of transcriptional regulatory modules reveals new insights into human gene expression Genome Res 2006 16 656 668 10.1101/gr.4866006 16606704 PMC1457048

[B26] Gupta M Liu JS *De novo cis*-regulatory module elicitation for eukaryotic genomes Proc Natl Acad Sci USA 2005 102 7079 7084 10.1073/pnas.0408743102 15883375 PMC1129096

[B27] Zhou Q Wong WH CisModule: *de novo *discovery of *cis*-regulatory modules by hierarchical mixture modeling Proc Natl Acad Sci USA 2004 101 12114 12119 10.1073/pnas.0402858101 15297614 PMC514443

[B28] Ooi L Wood IC Chromatin crosstalk in development and disease: lessons from REST Nat Rev Genet 2007 8 544 554 10.1038/nrg2100 17572692

[B29] Tsai MC Manor O Wan Y Mosammaparast N Wang JK Lan F Shi Y Segal E Chang HY Long noncoding RNA as modular scaffold of histone modification complexes Science 2010 329 689 693 10.1126/science.1192002 20616235 PMC2967777

[B30] Li T Hu JF Qiu X Ling J Chen H Wang S Hou A Vu TH Hoffman AR CTCF regulates allelic expression of *Igf2 *by orchestrating a promoter-polycomb repressive complex 2 intrachromosomal loop Mol Cell Biol 2008 28 6473 6482 10.1128/MCB.00204-08 18662993 PMC2577414

[B31] Mikkelsen TS Ku M Jaffe DB Issac B Lieberman E Giannoukos G Alvarez P Brockman W Kim TK Koche RP Lee W Mendenhall E O'Donovan A Presser A Russ C Xie X Meissner A Wernig M Jaenisch R Nusbaum C Lander ES Bernstein BE Genome-wide maps of chromatin state in pluripotent and lineage-committed cells Nature 2007 448 553 560 10.1038/nature06008 17603471 PMC2921165

[B32] Koch CM Andrews RM Flicek P Dillon SC Karaöz U Clelland GK Wilcox S Beare DM Fowler JC Couttet P James KD Lefebvre GC Bruce AW Dovey OM Ellis PD Dhami P Langford CF Weng Z Birney E Carter NP Vetrie D Dunham I The landscape of histone modifications across 1% of the human genome in five human cell lines Genome Res 2007 17 691 707 10.1101/gr.5704207 17567990 PMC1891331

[B33] Kim TK Hemberg M Gray JM Costa AM Bear DM Wu J Harmin DA Laptewicz M Barbara-Haley K Kuersten S Markenscoff-Papadimitriou E Kuhl D Bito H Worley PF Kreiman G Greenberg ME Widespread transcription at neuronal activity-regulated enhancers Nature 2010 465 182 187 10.1038/nature09033 20393465 PMC3020079

[B34] Zentner GE Tesar PJ Scacheri PC Epigenetic signatures distinguish multiple classes of enhancers with distinct cellular functions Genome Res 2011 21 1273 1283 10.1101/gr.122382.111 21632746 PMC3149494

[B35] MacQuarrie KL Fong AP Morse RH Tapscott SJ Genome-wide transcription factor binding: beyond direct target regulation Trends Genet 2011 27 141 148 10.1016/j.tig.2011.01.001 21295369 PMC3068217

[B36] Creyghton MP Cheng AW Welstead GG Kooistra T Carey BW Steine EJ Hanna J Lodato MA Frampton GM Sharp PA Boyer LA Young RA Jaenisch R Histone H3K27ac separates active from poised enhancers and predicts developmental state Proc Natl Acad Sci USA 2010 107 21931 21936 10.1073/pnas.1016071107 21106759 PMC3003124

[B37] Kanhere A Viiri K Araújo CC Rasaiyaah J Bouwman RD Whyte WA Pereira CF Brookes E Walker K Bell GW Pombo A Fisher AG Young RA Jenner RG Short RNAs are transcribed from repressed polycomb target genes and interact with polycomb repressive complex-2 Mol Cell 2010 38 675 688 10.1016/j.molcel.2010.03.019 20542000 PMC2886029

[B38] Marson A Levine SS Cole MF Frampton GM Brambrink T Johnstone S Guenther MG Johnston WK Wernig M Newman J Calabrese JM Dennis LM Volkert TL Gupta S Love J Hannett N Sharp PA Bartel DP Jaenisch R Young RA Connecting microRNA genes to the core transcriptional regulatory circuitry of embryonic stem cells Cell 2008 134 521 533 10.1016/j.cell.2008.07.020 18692474 PMC2586071

[B39] Guttman M Amit I Garber M French C Lin MF Feldser D Huarte M Zuk O Carey BW Cassady JP Cabili MN Jaenisch R Mikkelsen TS Jacks T Hacohen N Bernstein BE Kellis M Regev A Rinn JL Lander ES Chromatin signature reveals over a thousand highly conserved large non-coding RNAs in mammals Nature 2009 458 223 227 10.1038/nature07672 19182780 PMC2754849

[B40] Visel A Blow MJ Li Z Zhang T Akiyama JA Holt A Plajzer-Frick I Shoukry M Wright C Chen F Afzal V Ren B Rubin EM Pennacchio LA ChIP-seq accurately predicts tissue-specific activity of enhancers Nature 2009 457 854 858 10.1038/nature07730 19212405 PMC2745234

[B41] Pennacchio LA Ahituv N Moses AM Prabhakar S Nobrega MA Shoukry M Minovitsky S Dubchak I Holt A Lewis KD Plajzer-Frick I Akiyama J De Val S Afzal V Black BL Couronne O Eisen MB Visel A Rubin EM *In vivo *enhancer analysis of human conserved non-coding sequences Nature 2006 444 499 502 10.1038/nature05295 17086198

[B42] Nobrega MA Ovcharenko I Afzal V Rubin EM Scanning human gene deserts for long-range enhancers Science 2003 302 413 10.1126/science.1088328 14563999

[B43] Heintzman ND Hon GC Hawkins RD Kheradpour P Stark A Harp LF Ye Z Lee LK Stuart RK Ching CW Ching KA Antosiewicz-Bourget JE Liu H Zhang X Green RD Lobanenkov VV Stewart R Thomson JA Crawford GE Kellis M Ren B Histone modifications at human enhancers reflect global cell-type-specific gene expression Nature 2009 459 108 112 10.1038/nature07829 19295514 PMC2910248

[B44] Gotea V Visel A Westlund JM Nobrega MA Pennacchio LA Ovcharenko I Homotypic clusters of transcription factor binding sites are a key component of human promoters and enhancers Genome Res 2010 20 565 577 10.1101/gr.104471.109 20363979 PMC2860159

[B45] Rada-Iglesias A Bajpai R Swigut T Brugmann SA Flynn RA Wysocka J A unique chromatin signature uncovers early developmental enhancers in humans Nature 2011 470 279 283 10.1038/nature09692 21160473 PMC4445674

[B46] Ravasi T Suzuki H Cannistraci CV Katayama S Bajic VB Tan K Akalin A Schmeier S Kanamori-Katayama M Bertin N Carninci P Daub CO Forrest AR Gough J Grimmond S Han JH Hashimoto T Hide W Hofmann O Kamburov A Kaur M Kawaji H Kubosaki A Lassmann T van Nimwegen E MacPherson CR Ogawa C Radovanovic A Schwartz A Teasdale RD An atlas of combinatorial transcriptional regulation in mouse and man Cell 2010 140 744 752 10.1016/j.cell.2010.01.044 20211142 PMC2836267

[B47] Vaquerizas JM Kummerfeld SK Teichmann SA Luscombe NM A census of human transcription factors: function, expression and evolution Nat Rev Genet 2009 10 252 263 10.1038/nrg2538 19274049

[B48] Rye MB Sætrom P Drabløs F A manually curated ChIP-seq benchmark demonstrates room for improvement in current peak-finder programs Nucleic Acids Res 2011 39 e25 10.1093/nar/gkq1187 21113027 PMC3045577

[B49] Kharchenko PV Tolstorukov MY Park PJ Design and analysis of ChIP-seq experiments for DNA-binding proteins Nat Biotechnol 2008 26 1351 1359 10.1038/nbt.1508 19029915 PMC2597701

[B50] Xu H Handoko L Wei XL Ye CP Sheng JP Wei CL Lin F Sung WK A signal-noise model for significance analysis of ChIP-seq with negative control Bioinformatics 2010 26 1199 1204 10.1093/bioinformatics/btq128 20371496

[B51] Wallerman O Motallebipour M Enroth S Patra K Bysani MS Komorowski J Wadelius C Molecular interactions between HNF4a, FOXA2 and GABP identified at regulatory DNA elements through ChIP-sequencing Nucleic Acids Res 2009 37 7498 7508 10.1093/nar/gkp823 19822575 PMC2794179

[B52] Wray GA Hahn MW Abouheif E Balhoff JP Pizer M Rockman MV Romano LA The evolution of transcriptional regulation in eukaryotes Mol Biol Evol 2003 20 1377 1419 10.1093/molbev/msg140 12777501

[B53] Lieberman-Aiden E van Berkum NL Williams L Imakaev M Ragoczy T Telling A Amit I Lajoie BR Sabo PJ Dorschner MO Sandstrom R Bernstein B Bender MA Groudine M Gnirke A Stamatoyannopoulos J Mirny LA Lander ES Dekker J Comprehensive mapping of long-range interactions reveals folding principles of the human genome Science 2009 326 289 293 10.1126/science.1181369 19815776 PMC2858594

[B54] Cuddapah S Jothi R Schones DE Roh TY Cui K Zhao K Global analysis of the insulator binding protein CTCF in chromatin barrier regions reveals demarcation of active and repressive domains Genome Res 2009 19 24 32 19056695 10.1101/gr.082800.108 PMC2612964

[B55] Kim TH Abdullaev ZK Smith AD Ching KA Loukinov DI Green RD Zhang MQ Lobanenkov VV Ren B Analysis of the vertebrate insulator protein CTCF-binding sites in the human genome Cell 2007 128 1231 1245 10.1016/j.cell.2006.12.048 17382889 PMC2572726

[B56] Bernstein BE Mikkelsen TS Xie X Kamal M Huebert DJ Cuff J Fry B Meissner A Wernig M Plath K Jaenisch R Wagschal A Feil R Schreiber SL Lander ES A bivalent chromatin structure marks key developmental genes in embryonic stem cells Cell 2006 125 315 326 10.1016/j.cell.2006.02.041 16630819

[B57] Pekowska A Benoukraf T Ferrier P Spicuglia S A unique H3K4me2 profile marks tissue-specific gene regulation Genome Res 2010 20 1493 1502 10.1101/gr.109389.110 20841431 PMC2963813

[B58] Boyle AP Davis S Shulha HP Meltzer P Margulies EH Weng Z Furey TS Crawford GE High-resolution mapping and characterization of open chromatin across the genome Cell 2008 132 311 322 10.1016/j.cell.2007.12.014 18243105 PMC2669738

[B59] Gaulton KJ Nammo T Pasquali L Simon JM Giresi PG Fogarty MP Panhuis TM Mieczkowski P Secchi A Bosco D Berney T Montanya E Mohlke KL Lieb JD Ferrer J A map of open chromatin in human pancreatic islets Nat Genet 2010 42 255 259 10.1038/ng.530 20118932 PMC2828505

[B60] Karolchik D Baertsch R Diekhans M Furey TS Hinrichs A Lu YT Roskin KM Schwartz M Sugnet CW Thomas DJ Weber RJ Haussler D Kent WJ University of California Santa Cruz The UCSC Genome Browser Database Nucleic Acids Res 2003 31 51 54 10.1093/nar/gkg129 12519945 PMC165576

[B61] Zhang Y Liu T Meyer CA Eeckhoute J Johnson DS Bernstein BE Nusbaum C Myers RM Brown M Li W Liu XS Model-based analysis of ChIP-Seq (MACS) Genome Biol 2008 9 R137 10.1186/gb-2008-9-9-r137 18798982 PMC2592715

[B62] Jothi R Cuddapah S Barski A Cui K Zhao K Genome-wide identification of *in vivo *protein-DNA binding sites from ChIP-Seq data Nucleic Acids Res 2008 36 5221 5231 10.1093/nar/gkn488 18684996 PMC2532738

[B63] Zang CZ Schones DE Zeng C Cui KR Zhao KJ Peng WQ A clustering approach for identification of enriched domains from histone modification ChIP-Seq data Bioinformatics 2009 25 1952 1958 10.1093/bioinformatics/btp340 19505939 PMC2732366

