# Supplementary material for: Clustered ChIP-Seq-defined transcription factor binding sites and histone modifications map distinct classes of regulatory elements
Source: BMC Biol. 2011 Nov 24;9:80. doi: 10.1186/1741-7007-9-80 (PMC3239327; doi:10.1186/1741-7007-9-80)
Supplement: Additional file 1 — Supplementary figures and Tables S1 to S7. [file 1741-7007-9-80-S1.PDF]

Supplementary Figure S1

Percentage overlap between individual transcription factors and annotated promoters/genes histone modifications, open chromatin and other transcription factors clusters for (A) K562 and (B) Gm12878 displayed as heatmaps. Most factors prefer regions of accessible chromatin marked by methylation of H3K4 and acetylation. The number of peaks identified for each transcription factor is included after each factor name. Note that the same factors do not necessarily have a similar number of peaks and overlap profiles in both cell-lines.

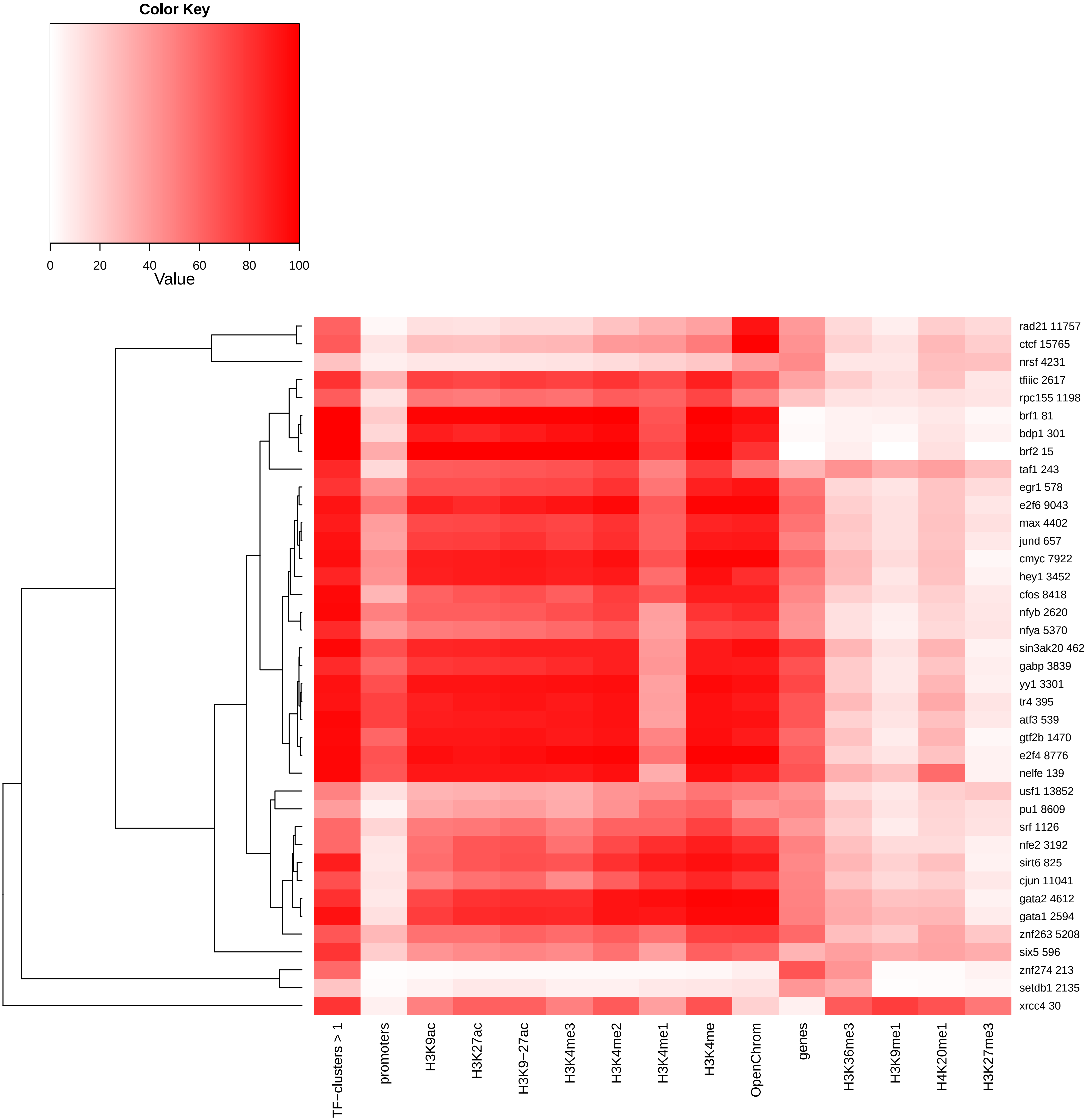

Color Key
0
20
40
60
80
100
Value
rad21 11757
ctcf 15765
nrsf 4231
tfiiic 2617
rpc155 1198
brf1 81
bdp1 301
brf2 15
taf1 243
egr1 578
e2f6 9043
max 4402
jund 657
cmyc 7922
hey1 3452
cfos 8418
nfyb 2620
nfya 5370
sin3ak20 462
gabp 3839
yy1 3301
tr4 395
atf3 539
gtf2b 1470
e2f4 8776
nelfe 139
usf1 13852
pu1 8609
srf 1126
nfe2 3192
sirt6 825
cjun 11041
gata2 4612
gata1 2594
znf263 5208
six5 596
znf274 213
setdb1 2135
xrcc4 30
TF−clusters > 1
promoters
H3K9ac
H3K27ac
H3K9−27ac
H3K4me3
H3K4me2
H3K4me1
H3K4me
OpenChrom
genes
H3K36me3
H3K9me1
H4K20me1
H3K27me3

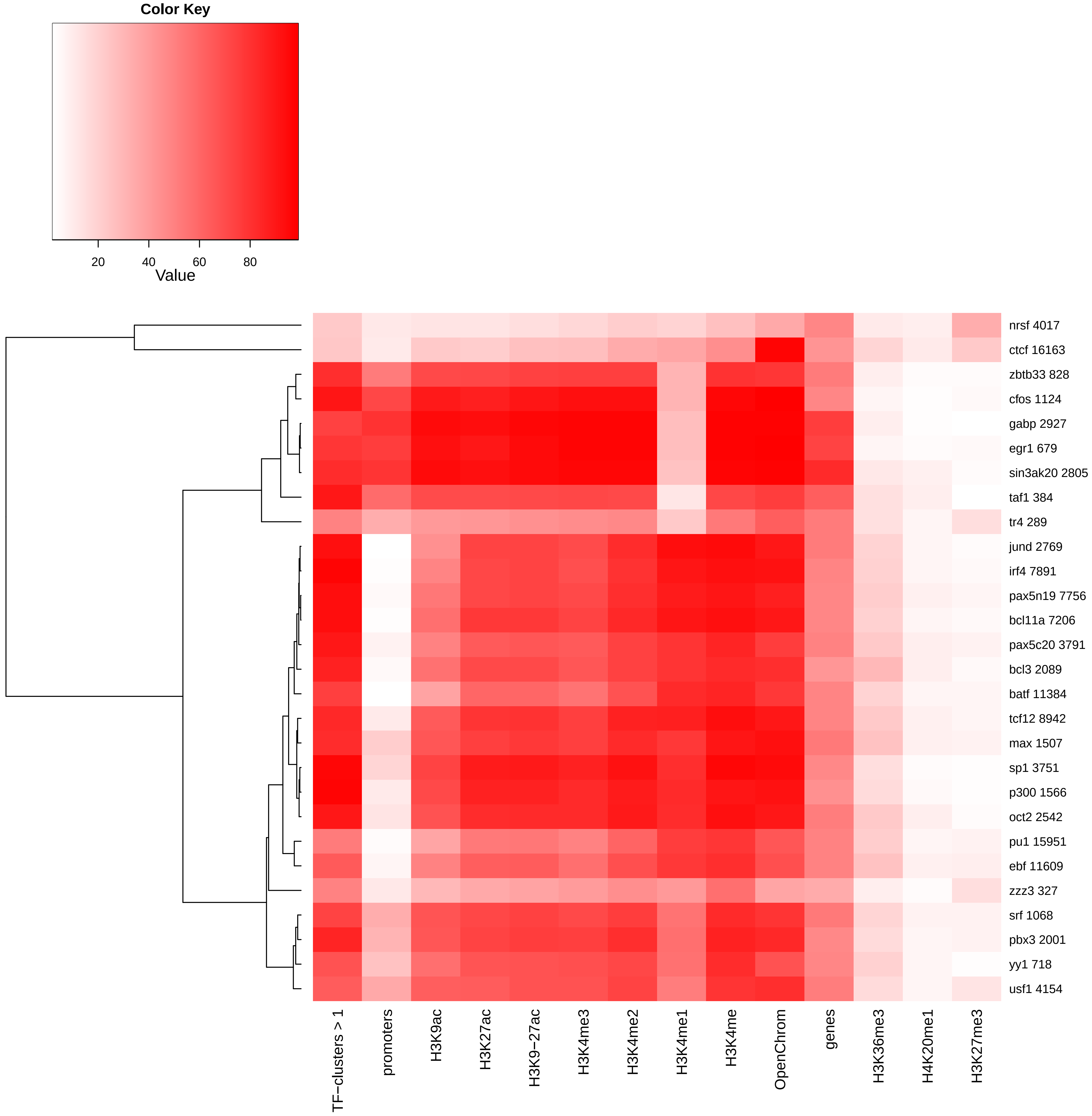

Color Key
20
40
60
80
Value
nrsf 4017
ctcf 16163
zbtb33 828
cfos 1124
gabp 2927
egr1 679
sin3ak20 2805
taf1 384
tr4 289
jund 2769
irf4 7891
pax5n19 7756
bcl11a 7206
pax5c20 3791
bcl3 2089
batf 11384
tcf12 8942
max 1507
sp1 3751
p300 1566
oct2 2542
pu1 15951
ebf 11609
zzz3 327
srf 1068
pbx3 2001
yy1 718
usf1 4154
TF−clusters > 1
promoters
H3K9ac
H3K27ac
H3K9−27ac
H3K4me3
H3K4me2
H3K4me1
H3K4me
OpenChrom
genes
H3K36me3
H4K20me1
H3K27me3

Supplementary Figure S2
Overlap between annotated genes, promoters and chromatin domains in (A) K562 and (B) Gm12878 displayed as a heatmap.

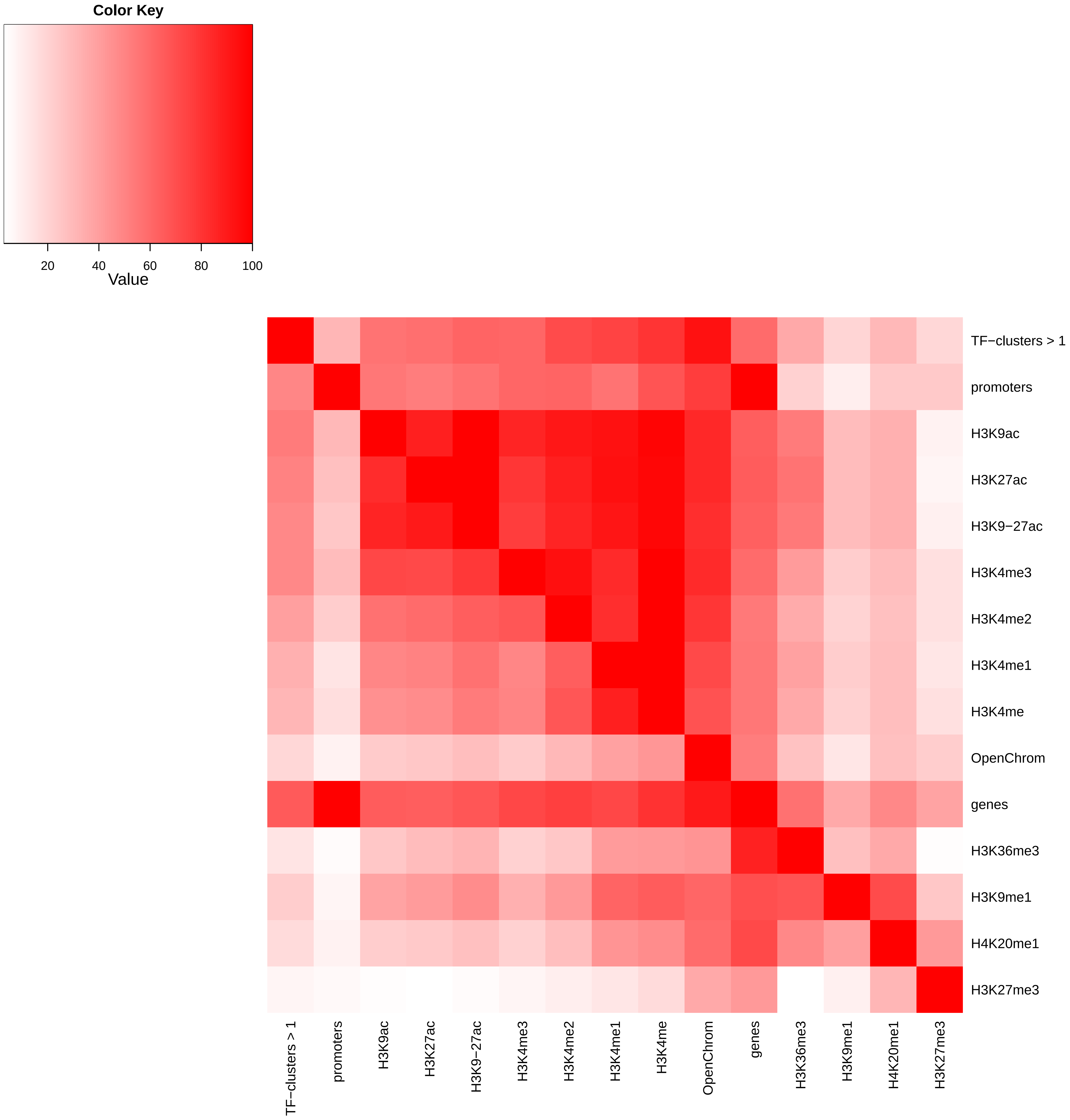
Color Key
20
40
60
80
100
Value
TF−clusters > 1
promoters
H3K9ac
H3K27ac
H3K9−27ac
H3K4me3
H3K4me2
H3K4me1
H3K4me
OpenChrom
genes
H3K36me3
H3K9me1
H4K20me1
H3K27me3
TF−clusters > 1
promoters
H3K9ac
H3K27ac
H3K9−27ac
H3K4me3
H3K4me2
H3K4me1
H3K4me
OpenChrom
genes
H3K36me3
H3K9me1
H4K20me1
H3K27me3

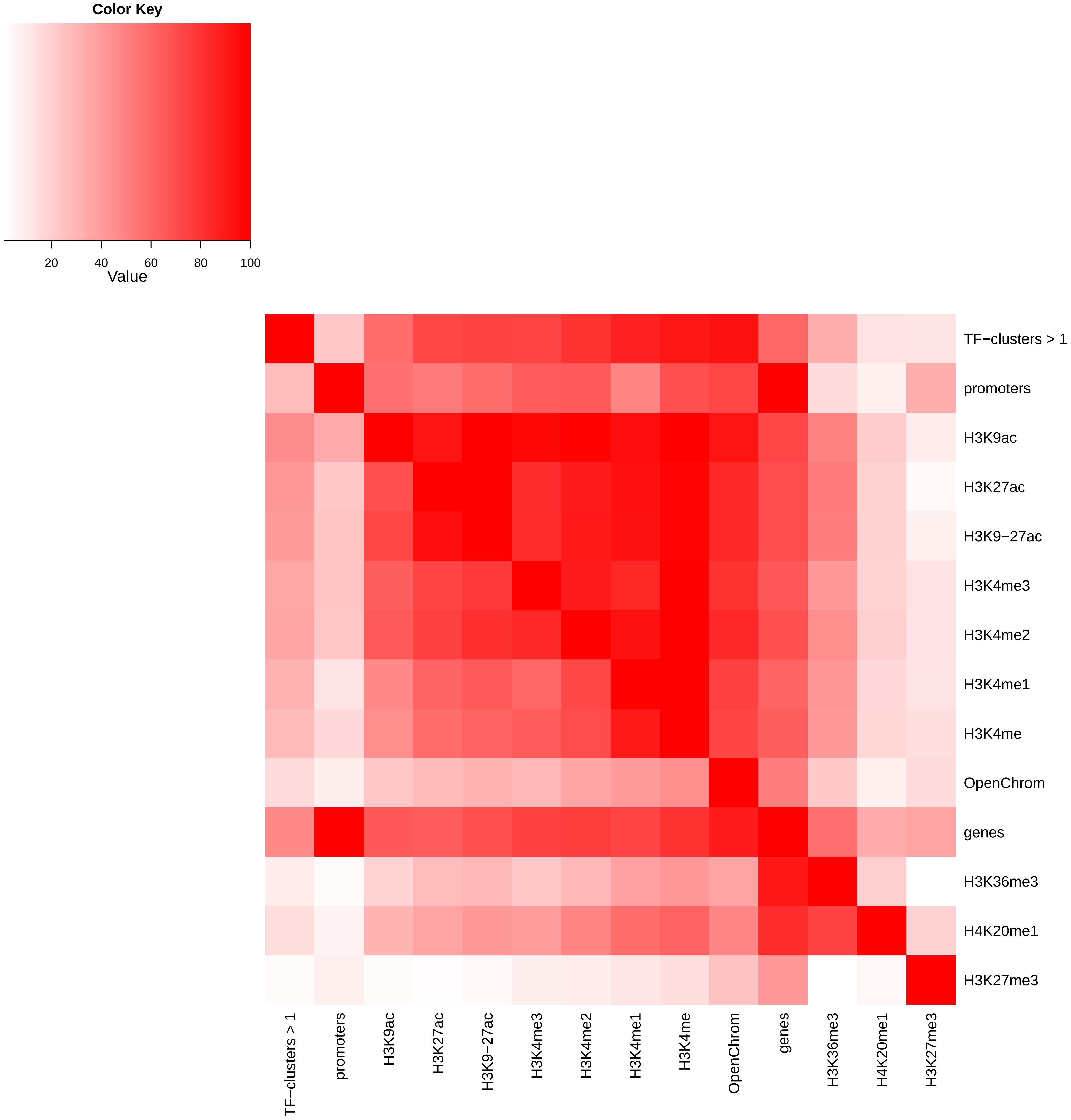

Color Key
20
40
60
80
100
Value
TF−clusters > 1
promoters
H3K9ac
H3K27ac
H3K9−27ac
H3K4me3
H3K4me2
H3K4me1
H3K4me
OpenChrom
genes
H3K36me3
H4K20me1
H3K27me3

Supplementary Figure S3
Profiles of histone modification and Pol2 around TSS for transcribed and silent genes in Gm12878.

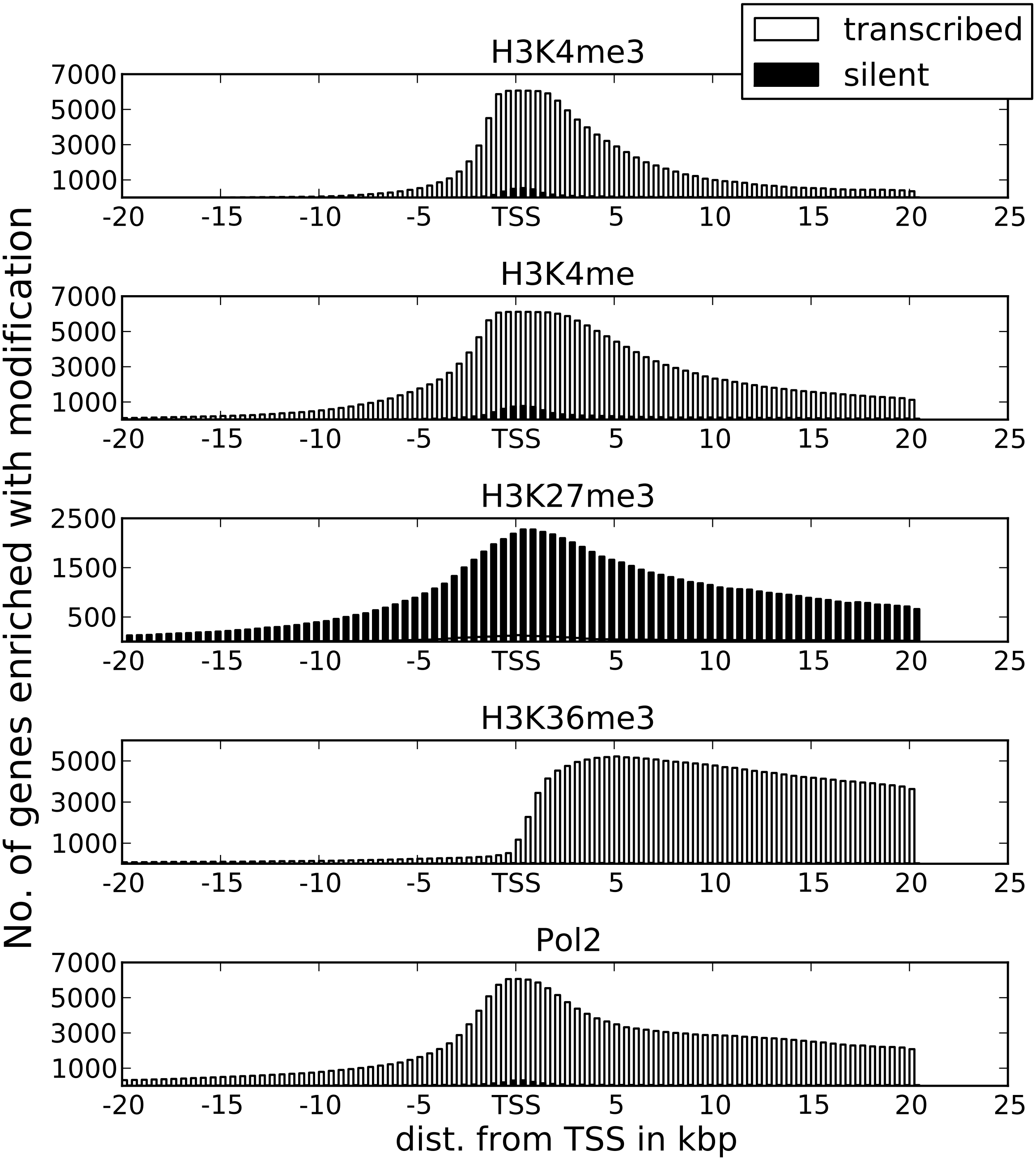

transcribed
silent
H3K4me3
H3K4me
H3K27me3
H3K36me3
Pol2
No. of genes enriched with modification
dist. from TSS in kbp
-20
-15
-10
-5
TSS
5
10
15
20
25
7000
5000
3000
1000
2500
1500
500

Supplementary Table S4: Different properties for promoter and gene clusters for (A) K562 and (B) Gm12878. Average bin scores was calculated by ranking the peak-sets for each transcription factor with respect to ChIP-Seq tag-intensity, and separate them into 20 bins with an equal number of peaks in each bin. A high average bin-score for a cluster means it consist of more highly ranked peaks.

A)

Expression	Clusters in..	No. of clusters	Most abundant TFs	Average No. of TFs in clusters	Avg. bin score of clusters
High	All genes and promoters	9486	E2F4, E2F6, C-FOS, C-MYC	4.3	11.5
	Promoters	5354 (56%)	E2F4, E2F6, C-MYC, YY1, GABP, C-FOS	5.2	12.2
	H3K4me	3549 (37%)	C-JUN, C-FOS, C-MYC, CTCF, GATA2, USF1	3.2	10.7
	Outside H3K4me and promoters	583 (6%)	CTCF, RAD21	2.2	10.4
Zero	All genes and promoters	3049	CTCF, RAD21, USF1, C-FOS, E2F4, C-JUN	2.9	10.6
	Promoters	590 (19%)	E2F6, CTCF, E2F4, C-FOS, USF1, RAD21	3.1	10.1
	H3K4me	1536 (50%)	CTCF, C-JUN, E2F6, C-FOS, RAD21, USF1	3.2	11.0
	Outside H3K4me and promoters	923 (30%)	CTCF, RAD21, USF1	2.2	10.0

B)

Expression	Clusters in..	No. of clusters	Most abundant TFs	Average No. of TFs in clusters	Significance of peaks in clusters
High	All genes and promoters	8067	TCF12, EBF, PU1, BATF, IRF4, PAX5n19	3.5	11.4
	Promoters	3252 (40%)	SIN3Ak20, GABP, USF1, TCF12	3.3	12.7
	H3K4me	4630 (57%)	BATF, PU1, EBF, IRF4, TCF12, PAX5n19	3.7	12.7
	Outside H3K4me and promoters	185 (2%)	CTCF, EBF, PAX5n19, PAX5c20	2.6	8.7
Zero	All genes and promoters	833	BATF, PU1, IRF4, PAX5n19	3.2	10.2
	Promoters	94 (11%)	CTCF, NRSF, PU1, SIN3Ak20	2.5	11.0
	H3K4me	530 (64%)	BATF, PU1, IRF4, PAX5n19	3.5	10.6
	Outside H3K4me and promoters	209 (25%)	CTCF, BATF, EBF, IRF4,PAX5n19	2.8	9.1

Supplementary Figure S5
Percentage of transcription-factor clusters occupied by individual factors in both cell lines. The factors are sorted from left to right according to their coverage in enhancer clusters.

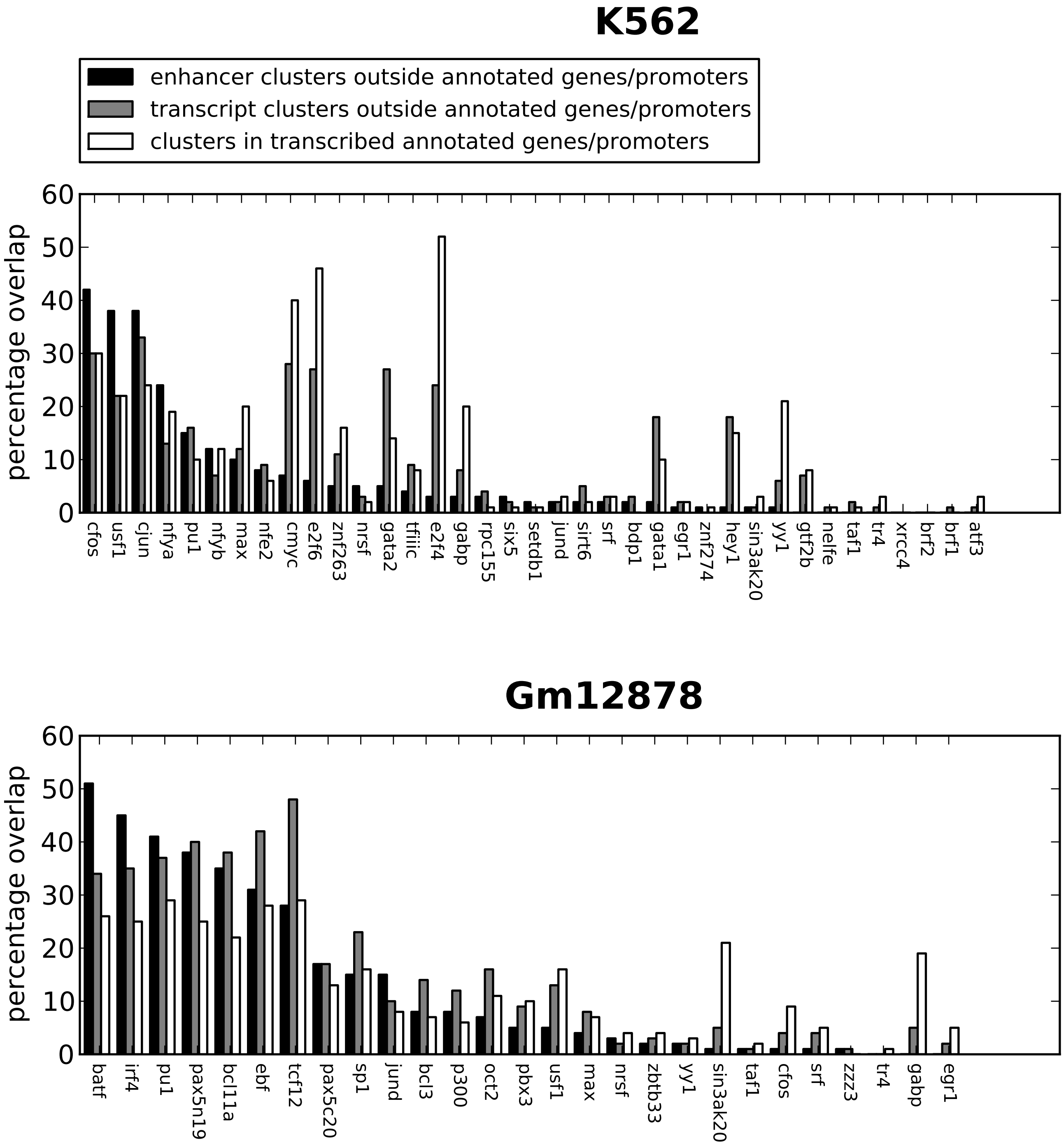
K562
enhancer clusters outside annotated genes/promoters
transcript clusters outside annotated genes/promoters
clusters in transcribed annotated genes/promoters
percentage overlap
0
10
20
30
40
50
60
cfos
usf1
cjun
nfya
pu1
nfyb
max
nfe2
cmyc
e2f6
znf263
nrsf
gata2
tfiiic
e2f4
gabp
rpc155
six5
setdb1
jund
sirt6
srf
bdp1
gata1
egr1
znf274
hey1
sin3ak20
yy1
gtf2b
nelfe
taf1
tr4
xrcc4
brf2
brf1
atf3
Gm12878
percentage overlap
0
10
20
30
40
50
60
batf
irf4
pu1
pax5n19
bcl11a
ebf
tcf12
pax5c20
sp1
jund
bcl3
p300
oct2
pbx3
usf1
max
nrsf
zbtb33
yy1
sin3ak20
taf1
cfos
srf
zzz3
tr4
gabp
egr1

Supplementary Figure S6

A modest selection of transcription factors shows good coverage of regulatory elements. (A). Coverage of annotated promoters grouped by gene expression when singleton peaks are included in addition to clusters. (B) Coverage of transcript and enhancer cluster regions using both singleton peaks and peak-clusters. (C) Coverage of transcript and enhancer clusters using only peak-clusters (more than one peak in each cluster).

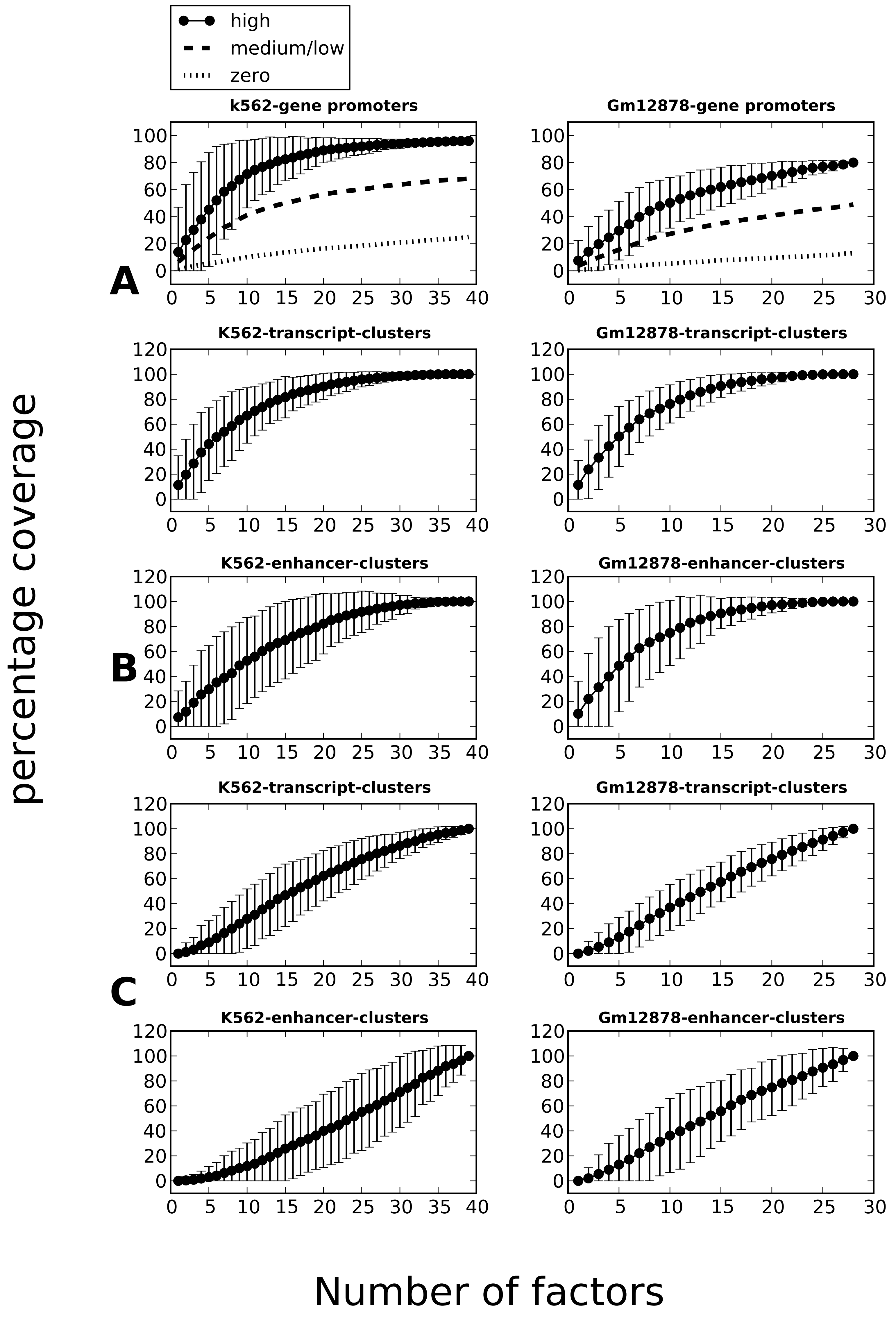
high
medium/low
zero
k562-gene promoters
Gm12878-gene promoters
K562-transcript-clusters
Gm12878-transcript-clusters
K562-enhancer-clusters
Gm12878-enhancer-clusters
K562-transcript-clusters
Gm12878-transcript-clusters
K562-enhancer-clusters
Gm12878-enhancer-clusters
A
B
C
percentage coverage
Number of factors

Supplementary Material S7

Downloaded data from ENCODE at the UCSC Genome Browser

Transcription factors

Yale/UC-Davis/Harvard:

ATF3	K562
BDP1	K562
BRF1	K562
BRF2	K562
C-FOS	Both
C-JUN	K562
C-MYC	K562
JUND	Both
E2F4	K562
E2F6	K562
GATA1	K562
GATA2	K562
GTF2B	K562
MAX	Both
NELFE	K562
NFE2	K562
NFYA	K562
NFYB	K562
RAD21	K562
RPC155	K562
SETDB1	K562
SIRT6	K562
TFIIIC	K562
TR4	Both
XRCC4	K562
YY1	Both
ZNF263	K562
ZNF274	K562
ZZZ3	Gm12878

HudsonAlpha Institute:

BATF	Gm12878
BCL11A	Gm12878
BCL3	Gm12878
EBF	Gm12878
EGR1	Both
GABP	Both
IRF4	Gm12878
HEY1	K562

NRSF	Both
PU1	Both
OCT2	Gm12878
p300	Gm12878
PAX5c20	Gm12878
PAX5n19	Gm12878
PBX3	Gm12878
SIN3A	Both
SIX5	K562
SP1	Gm12878
SRF	Both
TAF1	Both
TCF12	Gm12878
USF1	Both
ZBTB33	Gm12878

University of Washington:

CTCF	Both

Histone Modifications

University of Washington

H3K36me3	Both
H3K4me3	Both
H3K27me3	Both

Broad Institute

H3K36me3	Both
H3K4me3	Both
H3K27me3	Both
H3K4me2	Both
H3K4me1	Both
H3K27ac	Both
H3K9ac	Both
H3K9me1	K562
H4K20me1	Both

RNA polymerase

Yale/UC-Davis/Harvard:

RNA Pol II	Both
RNA Pol III	Both

HudsonAlpha Institute:

RNA Pol II	Both

Broad Institute:

RNA Pol IIb

Open Chromatin

Duke/UNC/UT

DNase HS	Both
FAIRE-Seq	Both

RNA-Seq

Caltech

RNA-Seq	Both

Downloaded data from other sources

Gene annotations

UCSC refGene. Download date 17.08.2010

miRNA

Dataset from Marson et al.

lincRNA

Human lincRNA based on the pipeline from Guttman et al.
